# PFAS-induced morpho-physiological, photosynthetic and tissue accumulation responses of pot-grown hemp, sunflower and maize under soil amendment with humic acids

**DOI:** 10.3389/fpls.2026.1791472

**Published:** 2026-07-07

**Authors:** Francesco Valente, Gilberto Chisini Granzotto, Anna Panozzo, Pranay Kumar Bolla, Antonio Masi, Patrizia Brunetti, Giuseppe Capobianco, Giovanna Visioli, Teofilo Vamerali

**Affiliations:** 1 Department of Agronomy, Food, Natural Resources, Animals and the Environment, University of Padua, Padua, Italy; 2 Research Institute on Terrestrial Ecosystems (IRET) – National Research Council of Italy (CNR), Rome, Italy; 3 Department of Chemical Engineering Materials and Environment, SAPIENZA – University of Rome, Rome, Italy; 4 Department of Chemistry, Life Sciences and Environmental Sustainability, University of Parma, Parma, Italy

**Keywords:** *Cannabis sativa*, *Helianthus annuus*, PFOA - perfluorooctanoic acid, PFOS - perfluorooctane sulfonic acid, phytoremediation, PSII efficiency, stomatal conductance, *Zea mays*

## Abstract

Perfluorooctanoic acid (PFOA) and perfluorooctane sulfonic acid (PFOS) are persistent per- and polyfluoroalkyl substances (PFAS) of environmental concern that can interfere with plant growth and physiological processes. This study evaluated the morpho-physiological response and PFAS accumulation in hemp (*Cannabis sativa* L.), maize (*Zea mays* L.), and sunflower (*Helianthus annuus* L.) grown in soil-filled pots for 32 days under greenhouse conditions. Silty loam soil was spiked with PFOA+PFOS (500 µg kg^-1^ each), with or without a single exploratory soil application of humic acids (HAs; 1 g kg^-1^). Across species, PFAS exposure caused no significant reduction in total biomass or visible phytotoxicity, and leaf SPAD values were largely maintained. Photosynthetic measurements were conducted exclusively on hemp, which revealed increased PSII operating efficiency (Fv′/Fm′) while significantly reducing stomatal conductance (−46%), transpiration (−40%), and net CO_2_ assimilation, alongside an 83% reduction in electron transport rate at near-saturating irradiance, under PFAS. This response is consistent with a decoupling between photochemistry and carbon assimilation, likely indicative of physiological stress. Hemp showed the highest foliar PFAS accumulation (PFOA: 5.39 µg g^-1^ d.w.; PFOS: 2.59 µg g^-1^ d.w.) and strong root−to−shoot translocation. Maize and sunflower achieved comparable or greater total plant PFAS burden per plant owing to their higher biomass. HAs significantly increased total dry weight in maize (+17%) and sunflower (+20%), and increased sunflower root dry weight (+49%). While the effects of HAs on PFAS accumulation were largely non-significant, in hemp PFAS+HAs significantly reduced the translocation factor (TF) of both PFOA and PFOS compared with PFAS alone. Future studies should evaluate PFAS removal over full growth cycles under field conditions, compare multiple HA rates and application methods including foliar spraying, and extend detailed photosynthetic characterization to all three species.

## 1 Introduction

Per- and polyfluoroalkyl substances (PFAS) are a class of chemical compounds widely used in industrial and consumer applications due to their resistance to heat, chemical degradation, and water-and-oil-repellent properties (Brunn et al., 2023). Their highly fluorinated carbon chain confers amphiphilic behavior. This affects their interactions with soil organic matter, root surfaces, and biological membranes, thereby affecting uptake, translocation, and plant physiological processes including water transport and photosynthesis (Banks et al., 1994; Costello and Lee, 2020; Blaine et al., 2014). Among PFAS, perfluorooctanoic acid (PFOA; C_8_HF_15_O_2_) and perfluorooctane sulfonic acid (PFOS; C_8_F_17_SO_3_H) are of particular concern because of their extensive use and persistence. Their different terminal groups, carboxyl in PFOA and sulfonic in PFOS, can influence environmental behavior and biological interactions (Buck et al., 2011; Scheringer et al., 2014).

Widespread PFAS use has resulted in ubiquitous environmental contamination, including in agricultural soils, through sources such as landfill leachate, aqueous film-forming foams, wastewater treatment plants, and fluorochemical manufacturing (Kavusi et al., 2023; Gebbink and van Leeuwen, 2020; Gallen et al., 2018). Elevated concentrations have been reported near firefighting and industrial sites, including 2.6 mg kg^-1^ of PFOS at a Norwegian firefighting training facility (Hale et al., 2017) and of Ʃ18PFAS up to 26.3 mg kg^-1^ in Chinese topsoil near perfluoro-octane-sulfonyl-fluoride (POSF) production plants (Jia et al., 2023; Gobelius et al., 2017; Ehsan et al., 2024). This is relevant for agriculture and public health because PFAS in soil can act as chronic chemical stressors, leach into water resources, enter crops and the food chain and bioaccumulate in living organisms (Costello and Lee, 2020).

PFAS exposure has been associated with cardiovascular disease, thyroid dysfunction, ulcerative colitis, and certain cancers (Biggeri et al., 2024). Their persistence in protein-rich tissues, including blood and liver, further underscores the importance of understanding PFAS transfer into crops and other biological systems (Brunn et al., 2023; Frisbee et al., 2009).

In plants, PFAS exposure can alter growth and metabolism, although visible phytotoxicity is not always observed at environmentally relevant concentrations (Costello and Lee, 2020). PFAS accumulation depends on factors such as chain length, soil texture, and soil organic matter content (Xu et al., 2022). PFAS, especially perfluoroalkyl acids (PFAAs), interact with soil organic matter through hydrophobic, electrostatic, and hydrogen bonding mechanisms that regulate their mobility, root bioavailability, and distribution within plant tissues (Mei et al., 2021; Costello and Lee, 2020). Long-chain PFAS such as PFOA and PFOS generally show stronger adsorption to soil organic matter, thereby limiting their bioavailability, whereas short-chain PFAS tend to be more mobile and readily translocated to shoots. Plant anatomical and physiological traits further modulate PFAS transport within the soil-plant continuum. The Casparian strip can restrict apoplastic flow and limit PFAS translocation to the shoot (Abou-Khalil et al., 2022; Blaine et al., 2014). Transpiration is a major driver of PFAS uptake and root-to-shoot translocation, linking contaminant accumulation to stomatal regulation, water transport, and leaf-level gas exchange (Blaine et al., 2014). Root anatomy also affects PFAS partitioning. Larger vascular cylinders may enhance shoot transport, whereas thicker taproots tend to retain long-chain molecules. Fine, elongated roots may favor short-chain PFAS translocation, while thick epidermis may impede initial uptake (Lesmeister et al., 2021; Blaine et al., 2014; Qian et al., 2023; He et al., 2023).

Experimental studies indicate that PFAS at environmentally relevant concentrations often induce compensatory physiological responses rather than acute phytotoxicity, although responses are species-dependent. For example, low PFAS concentrations increased stomatal conductance and net photosynthesis in hydroponically grown willow, possibly reflecting compensatory stress responses (Sharma et al., 2020; Karamat et al., 2024). However, emerging molecular evidence demonstrates that the absence of visible phytotoxicity does not preclude underlying cellular stress. Luche et al. (2026a) demonstrated that in hemp PFOA and PFOS inhibited the activity of key antioxidant enzymes, including ascorbate peroxidase (APX) and catalase (CAT), and promoted oxidative DNA damage despite the absence of macroscopic morphological symptoms. These findings suggest that apparent PFAS tolerance may mask underlying physiological and biochemical constraints that could affect long-term plant viability.

Although PFAS accumulation in plants raises food safety concerns, it also has stimulated interest in phytoremediation as a potential long-term strategy for the management of PFAS-contaminated soils and water (Beškoski et al., 2024; Cunningham et al., 1996). Previous studies have documented pronounced species-specific differences in PFAS uptake and partitioning. Some species show high shoot accumulation, whereas others achieve greater total PFAS removal mainly because of higher biomass production, indicating that tissue concentration and per-plant contaminant burden do not necessarily coincide (Huff et al., 2020; He et al., 2023). For example, He et al. (2023) reported bioconcentration factor (BCF) values above 10 for PFOS in *Festuca rubra* and removal efficiencies ranging from 0.04% to 41.4% across weed species. In the same study, *Juncus effusus* showed the greatest PFAS mass uptake because of its high biomass, while short-chain PFAS were generally more mobile and readily accumulated than long-chain compounds (He et al., 2023). In biomass crops such as hemp, mustard, and sunflower, microbial inoculation and fertilization have also been reported to influence PFAS accumulation, indicating that soil conditions can modify uptake and partitioning (Nassazzi et al., 2023). Phytoremediation offers advantages in sustainability and ecological integration, but its effectiveness remains limited by low removal efficiencies and PFAS retention in soil or leaching (Eun et al., 2022; Qi et al., 2022).

Among biomass crops, hemp (*Cannabis sativa* L.), maize (*Zea mays* L.), and sunflower (*Helianthus annuus* L.) provide a biologically meaningful comparison because they differ in photosynthetic pathway, biomass production, transpiration behavior, root architecture, and other traits relevant to PFAS transport (Nassazzi et al., 2023; He et al., 2023). Hemp has attracted attention for both high foliar PFAS accumulation and root-to-shoot translocation. Its deep taproot, high leaf area, and Type I primary cell walls may further favor transpiration-driven PFAS movement to aboveground tissues (Nassazzi et al., 2023; Luche et al., 2026a). Maize, by contrast, is a high-biomass C4 monocot with phenolic-rich Type II cell walls and a well-developed Casparian strip, traits that may restrict apoplastic transport and contribute to lower shoot concentrations even when total PFAS burden per plant is high owing to great biomass (Blaine et al., 2014; Nassazzi et al., 2023). Sunflower represents an intermediate case: it is a C3 dicot with high canopy transpiration and Type I cell walls similar to hemp, but with different root architecture and biomass partitioning that may lead to distinct uptake and translocation patterns (Nassazzi et al., 2023; He et al., 2023). Together, the combination of cell-wall type (Type I versus Type II), Casparian strip and vascular cylinder anatomy, leaf area, canopy transpiration, and root architecture provide a multi−trait mechanistic framework for comparing PFAS transport and partitioning. This framework clarifies how anatomical and physiological differences influence contaminant movement that extends the comparison beyond a simple C3 vs. C4 photosynthetic classification. Together, these species span the C3-C4 divide, the monocot-dicot anatomical distinction, and a broad biomass range potential, making them suitable for testing how species-specific traits shape PFAS accumulation and partitioning under soil exposure.

Humic acids (HAs) are high-molecular-weight fractions of humic substances formed during the decomposition of organic matter, characterized by various functional groups, particularly carboxyl and phenolic moieties (Stevenson, 1994). In plants, humic substances are known to exert biostimulant-like effects, including stimulation of root growth and nutrient uptake (Nardi et al., 2002; Canellas and Olivares, 2014; Muscolo et al., 2013). In PFAS-contaminated systems, HAs may interact with PFAS through competing mechanisms: PFAS-DOM complexation can reduce the freely available fraction through hydrophobic and electrostatic associations, while competition for sorption sites may alter adsorption-desorption dynamics and mobility in the soil matrix (Mei et al., 2021; Qi et al., 2022). At the same time, HA application may stimulate root growth, nutrient acquisition, and transpiration, which could indirectly enhance mass-flow PFAS uptake by plants (Canellas and Olivares, 2014). Liu et al. (2023) also demonstrated in hydroponically grown wheat that humic acids can modify PFAS transmembrane transport via slow-type anion channels, suggesting that HAs effects may extend beyond soil-phase interactions to root-level processes. The net outcome of these competing processes remains unresolved and likely depends on PFAS chemistry, soil properties, HA dose and application mode, and plant species (Qi et al., 2022; Liu et al., 2023).

Despite growing evidence that PFAS can accumulate in crops, the relationships among contaminant uptake, morpho-physiological responses, and photosynthetic performance under soil exposure remain poorly understood. In particular, it is unclear whether PFAS-induced limitations in net CO_2_ fixation arise mainly from stomatal constraints, biochemical constraints on carbon fixation, or alterations in photochemical energy partitioning, and whether these responses differ between species with contrasting photosynthetic pathways and morpho-anatomical traits. Existing soil-based multi-species PFAS studies have mainly focused on accumulation patterns and phytoextraction potential, predominantly on C3 species, with limited integration of detailed photosynthetic physiology and humic acid (HA) amendment effects under comparable experimental conditions (Blaine et al., 2014; He et al., 2023; Nassazzi et al., 2023).

In this context, the present study combines Liquid chromatography-tandem mass spectrometry (LC-MS/MS) quantification of PFOA and PFOS in plant tissues with a comparative analysis of morpho-physiological responses in three agronomically relevant crops grown under identical contaminated conditions, with or without a humic-acid soil amendment. Here, detailed photosynthetic assessment was preliminarily performed in hemp, while growth, chlorophyll-related traits, and PFAS partitioning were compared across hemp, sunflower, and maize. The inclusion of two C3 species and one C4 species allows evaluation of species-specific PFAS partitioning, root-to-shoot translocation, and the potential role of humic acids in shaping plant performance and contaminant distribution within the soil-plant system. To our knowledge, no published soil-based pot study has simultaneously evaluated a C3 dicot, a C4 monocot, and a second C3 dicot for PFAS uptake and humic acid amendment effects under identical conditions.

Based on these knowledge gaps, this study aimed to evaluate PFAS-induced morpho-physiological responses in hemp (*Cannabis sativa* L.), maize (*Zea mays* L.), and sunflower (*Helianthus annuus* L.) grown in soil artificially contaminated with combined PFOA and PFOS. The study also evaluates the influence of soil-applied humic acids on plant physiological performance and PFAS accumulation patterns, including possible effects on plant growth, PFAS uptake, and root-to-shoot partitioning. Specifically, the effects of PFAS exposure, with or without humic acids, were assessed in terms of *i*) plant growth and biomass allocation in hemp, maize, and sunflower; *ii*) leaf chlorophyll-related traits in all three species; *iii*) detailed gas-exchange and chlorophyll-fluorescence responses in hemp, as a species selected for in-depth physiological characterization; and *iv*) PFAS uptake and tissue-specific accumulation patterns in all three species.

## 2 Materials and methods

### 2.1 Experimental design, substrate preparation, and sowing

The trial was conducted in a greenhouse at the “Lucio Toniolo” experimental farm, University of Padua, Legnaro, Italy (45°21’ N, 11°57’ E), from 9 May to 10 June 2024 (32 days). The greenhouse was maintained at a day/night temperature of 25/18 °C, and a relative humidity of 60–70%. Plants were grown under the natural photoperiod with no supplementary artificial lighting. The natural daylength increased progressively from the 14 h 40 min at the start to 15 h 36 min at harvest.

The plant species considered in the study were hemp (*Cannabis sativa* L., monoecious seed variety “Earlina”; SOC, France), maize (*Zea mays* L., hybrid P0943, FAO class 500; Corteva Agriscience, Italy), and sunflower (*Helianthus annuus* L., hybrid P64HE144, Corteva Agriscience, Italy). The experimental setup followed a completely randomized design, comprising 48 pots randomly arranged on benches to ensure consistent growing conditions. Four treatments were applied: (i) Control (named CTRL) with no soil PFAS contamination and no humic acids application; (ii) Soil application of humic acids (named HAs); (iii) Soil PFAS treatment with perfluorooctanoic acid (PFOA) together with perfluorooctanesulfonate (PFOS); and (iv) Soil application of both PFOA+PFOS and humic acids (named PFAS+HAs). Species and treatment abbreviations are defined in **Table 1**.

**Table 1:** List of treatments and their abbreviations as a combination of three plant species and four soil managements.

Species	Treatment			
	Control	Humic acids	PFOA+PFOS	PFOA+PFOS+Humic acids
*Cannabis sativa* L. (CS)	CS + CTRL	CS + HAs	CS + PFAS	CS + PFAS + HAs
*Zea mays* L. (ZM)	ZM + CTRL	ZM + HAs	ZM + PFAS	ZM + PFAS + HAs
*Helianthus annuus* L. (HA)	HA + CTRL	HA + HAs	HA + PFAS	HA + PFAS + HAs

For hemp, six replicate pots were assigned to each treatment (n = 6). For sunflower and maize, three replicate pots were assigned to each treatment (n = 3), resulting in 48 pots in total, with pot as the experimental unit throughout the study. A higher number of replicates was used for hemp because of its greater variability during emergence and early vegetative development, including differences in seedling vigor and plant morphology, compared to maize and sunflower. The larger replication for hemp was adopted to better capture this within-species variability and improved statistical robustness for this species. Maize and sunflower, showing more uniform early growth as hybrids, were included primarily as comparative species for biomass production and PFAS accumulation.

Growth substrate was prepared by thoroughly mixing dry silty-loam soil collected from the “Lucio Toniolo” experimental farm (**Supplementary Table 1**) and river sand in a 1:1 weight ratio. The main soil characteristics were: pH 8.15, organic matter content 1.77%, N content 0.11%, and CEC 15.4 cmol(+) kg^–1^. To enrich the mixture, 56 g of NPK fertilizer (15-15-15) per 100 kg of substrate were added. This corresponded to 300 kg ha^-1^ of nitrogen (N), phosphorus (P_2_O_5_), and potassium (K_2_O), assuming a 25-cm substrate depth. NPK granules were first ground using a mortar and pestle to ensure uniformity, and the powder was homogenized with the substrate using an IMER Syntesi 300 (IMER group, Siena, IT) concrete mixer. Each pot had a ~28 cm height, 1.7 L volume, and was filled with 2 kg of substrate.

Hemp seeds were sown at a 0.5 cm depth, with 15 seeds per pot, and seedlings were thinned to 5 plants per pot at 7 DAS. Maize and sunflower were sown at 3 cm depth with 6 seeds per pot, aiming for 3 plants per pot after thinning. Final plant number per pot was established according to species-specific establishment patterns and growth habit in field cultivation, which require higher plant densities in hemp than maize and sunflower. Consequently, per-plant biomass and PFAS burden values should be interpreted within the context of these pot conditions, because the differing plant numbers among species may have affected within-species competition.

Irrigation was applied using a graduated beaker to ensure equal water volume distribution across replicate pots of the same species. Soil moisture was maintained near field capacity by daily irrigation, with water input adjusted according to pot weight losses due to evapotranspiration. The pots had no drainage holes at the bottom to prevent PFAS leaching; irrigation was managed to avoid waterlogging.

All experimental work involving PFOA and PFOS followed institutional safety protocols and national regulations for hazardous chemicals.

### 2.2 Treatments application

#### 2.2.1 PFAS treatment

On 17 May (8 DAS), an aqueous solution containing PFOA and PFOS was applied via irrigation to contaminate the soil. In the laboratory, 30 mg each of pure PFOA and PFOS were dissolved in 300 mL of deionized water by sonication (ELMASONIC S 100), yielding a 100 mg L^-1^ stock solution. Subsequently, 10 mL of this solution was pipetted into each pot to achieve a nominal concentration of 500 µg kg^–1^ per compound (1 mg kg^–1^ total) with 2 kg of substrate. All soil exposure values reported in this study refer to the nominal spiked concentrations at the time of application.

The selected PFOA and PFOS concentrations were based on previous controlled pot studies investigating PFAS accumulation and plant physiological responses in biomass crops (Nassazzi et al., 2023). These levels were considered sufficiently high to allow quantification of PFAS accumulation and translocation while avoiding severe phytotoxicity, thereby enabling the assessment of sub-lethal physiological responses. The concentration is also consistent with the upper range of soil contamination reported at PFAS-impacted sites associated with firefighting activities and industrial emissions (Hale et al., 2017; Ehsan et al., 2024). The combined application of PFOA and PFOS at the same concentration allowed comparison of plant responses to two structurally distinct long-chain PFAS, differing in functional group chemistry (carboxylate vs. sulfonate), under identical soil conditions.

#### 2.2.2 Soil application with humic acids

On 23 May (14 DAS), humic acids were applied to the soil using the commercial liquid product Humic Super bio (Tiller s.r.l., Pescantina, Verona, Italy), a liquid leonardite-derived humic formulation containing 12% w/w humic acids, obtained using potassium hydroxide (KOH) as extracting agent. Because the commercial formulation was produced by alkaline extraction, the treatment should be interpreted as the application of a humic extract formulation rather than a chemically pure humic-acid-only input. In the laboratory, 400 g of the formulation was dissolved with water to a final volume of 2 L, stirred for 5 min at 70 rpm, with a magnetic stirrer. The solution was then distributed evenly among the 24 pots assigned to HAs and PFAS + HAs treatments. This corresponded to 83.3 mL of solution per pot, and supplied 2 g humic acid per pot, equivalent to 1 g kg^-1^ of soil (0.1% w/w).

The application rate used in this study was selected as a single exploratory soil dose. It was based on previous pot experiments with leonardite-derived humic material showing that 1 g kg^−1^ soil was an effective soil application rate, and produced the highest plant biomass along with increased specific root length and nutrient uptake (Katkat et al., 2009; Bandiera et al., 2009). Given that the physiological activity of humic substances is concentration and source dependent (Nardi et al., 2002), a single HA dose was employed in this initial characterization experiment to determine the direction of HA effects on PFAS uptake, partitioning and plant physiology before future dose-response evaluation.

Humic acids were applied at 14 DAS, after seedling emergence and thinning, to impose the amendment under post-emergence conditions and avoid confounding effects on germination and very early establishment. Because PFAS were applied earlier, at 8 DAS, the PFAS+HAs treatment did not represent simultaneous co-application, and early PFAS soil-root interactions occurred before HA addition. The combined treatment should therefore be interpreted as sequential amendment of an already PFAS-exposed system.

### 2.3 Plant growth and physiological measurements

Vegetative parameters were monitored every 3–4 days from 11 DAS (20 May) to harvest, 32 DAS (10 June). The assessed parameters included plant height and SPAD (Soil Plant Analysis Development) index. Plant height was measured using a ruler from the stem base up to the last visible collar in maize, and up to the node bringing the highest leaf in hemp and sunflower. Measurements taken on 11 DAS (20 May) and 14 DAS (23 May) reflected only the effects of PFAS, as humic acids were applied on 23 May. Therefore, treatment comparisons involving humic acids are based on data collected from 18 DAS (27 May) onward.

The SPAD index, which indirectly assessed the leaf chlorophyll content, was measured using a handheld SPAD-502 equipment meter (Minolta, Japan). In maize, two readings were taken at 1/3 and 2/3 of the length of the youngest fully expanded leaf on each plant and then averaged across the three plants per pot. In hemp and sunflower, SPAD was measured in leaves at the penultimate (or last, if sufficiently developed) node. Five readings were taken in hemp (one per plant) and averaged to obtain one value per pot. In sunflower, SPAD was measured on opposite leaves of the first node, and subsequently on the two opposite leaves of the second node. Readings were taken from all the three plants within a pot and then averaged to obtain a pot-level value. SPAD measurements allowed to assess the effects of treatments within the same species but, because sampling positions and reading protocols differed among species to match species-specific leaf anatomy, cross-species comparisons of SPAD values should be made cautiously.

Gas-exchange and chlorophyll fluorescence were measured exclusively on hemp on 4 June (26 DAS), during the vegetative phase, using a LI-6800 Portable Photosynthetic System equipped with the 6800-01A fluorometer head (Li-COR Biosciences Inc., Lincoln, Nebraska, USA). Because measurements were collected at a single time point, they should be interpreted as a physiological snapshot rather than as a time-resolved characterization of PFAS-induced responses. The measured parameters included photosystem II (PSII) operating efficiency (*Fv*′/*Fm*′), stomatal conductance to water vapor (gsw), transpiration rate (E), net CO_2_ assimilation (A), non-photochemical quenching (NPQ), leaf temperature, and electron transport rate (ETR). All fluorescence parameters were recorded under light-adapted steady-state conditions using the multiphase flash protocol; Fv′/Fm′ therefore represents the light-adapted PSII operating efficiency rather than the dark-adapted maximum quantum efficiency (Fv/Fm). Fluorometer settings were as follows: block temperature 25 °C, reference CO_2_ 400 µmol mol^-1^, chamber air flow 500 µmol s^-1^, chamber relative humidity 65%, and leaf area aperture 6 cm².

Full light-response gas-exchange measurements were conducted exclusively on hemp, given its central role and recognized potential for the phytoextraction and accumulation of per- and polyfluoroalkyl substances, and the importance of fully characterizing its physiological performance (Nason et al., 2024). Each measurement sequence comprising an eight−step Photosynthetic Photon Flux Density (PPFD) ramp required approximately 15–20 minutes per plant, including leaf stabilization under tightly controlled chamber conditions. To minimize temporal and microclimatic variability during these full-curve measurements, gas-exchange assessments were prioritized for this species. In addition, hemp is a C3 dicot, and detailed light-response curves are most directly interpretable within the C3 biochemical framework of carbon fixation (Farquhar et al., 1980). Extending equivalent full-curve measurements to maize (a C4 species), although valuable, would have required separate parameterization that was outside the scope of this initial characterization.

For each replicate pot, photosynthetic measurements were obtained along a descending PPFD ramp comprising eight visible irradiance levels (1500, 1200, 900, 600, 300, 150, 50, and 0 µmol m^-2^ s^-1^). Measurements were taken on the topmost fully expanded leaf of one representative plant per pot, with six replicate pots per treatment (Valente et al., 2024). Gas-exchange and fluorescence measurements were used to provide species-specific mechanistic information for hemp and were not intended to support direct physiological comparisons among species. Cross-species comparisons, particularly between the C3 species (hemp, sunflower) and the C4 species (maize), were therefore limited to growth traits and PFAS accumulation metrics.

Accordingly, Fv′/Fm′, net CO_2_ assimilation (A), and NPQ are presented as PPFD-dependent response curves, while stomatal conductance (gsw), transpiration rate (E), and leaf temperature are reported as means averaged across the eight applied PPFD levels. ETR values at 1200 µmol m^-2^ s^-1^ (near saturating irradiance) were selected to highlight treatment-dependent differences in photochemical capacity and photoprotective responses (Huang et al., 2021).

The pots were transparent polyethylene terephthalate (PET) containers wrapped in aluminum foil to prevent algal growth and root exposure to light. PET containers were selected because recent studies have shown lower PFAS adsorption and analyte losses in PET relative to other commonly used plastic materials, helping to minimize potential container-induced bias during PFAS exposure experiments (Zenobio et al., 2022; Folorunsho et al., 2024). A visual assessment of the plants was also conducted to compare treatments at the end of the trial to complement analytical findings.

### 2.4 Dry biomass and PFOA and PFOS quantifications

Harvesting was carried out on 10 June (32 DAS). Plants were cut at the base, and aerial biomass was separated into leaves and stems. Roots were recovered from the soil by hydraulic sieving-centrifugation using a 500-μm mesh sieve. Leaves, stems, and roots were oven dried at 65 °C for 65 hours and subsequently weighed to determine their dry weight (d.w.).

For PFOS and PFOA analysis, dried tissue samples were ground to a fine powder using a Retsch GM 200 Mill (Retsch, Düsseldorf, Germany). Extractions were performed on 0.5 g d.w. samples using an accelerated solvent extraction system (Dionex ASE 350, Thermo Fisher Scientific, USA) with 15 mL of pure methanol at 125 °C and 9.0-10.3 MPa pressure. Extracts were filtered through a 0.22-μm CA membrane filter, transferred to Falcon tubes, and stored at 4 °C until analysis. PFAS concentrations were determined by LC-MS/MS using a triple quadrupole mass spectrometer (TSQ Quantiva, Thermo Fisher Scientific, USA) coupled with ultra-high performance liquid chromatography (Ultimate 3000 UHPLC, Dionex, Thermo Fisher Scientific, USA), following the method outlined by Sharma et al. (2020). Prior to analysis, a ^13^C-labelled PFOA and PFOS internal standard mixture (Wellington Laboratories, Canada) was added to the samples at a final concentration of 2 μg L^-1^. Samples were diluted with water before injection at 1:10 for stems, and 1:20 for leaves and roots. The instrument was operated in selected reaction monitoring (SRM) mode; raw data were processed using Skyline MS v. 21.2.0.425 (Adams et al., 2020) to quantify PFAS concentrations in plant tissues.

To ensure analytical quality, materials potentially containing fluoropolymers were avoided throughout the procedure. Method blanks, including extraction buffer and representative control-plant samples, were processed and analyzed as unknowns to monitor contamination introduced during extraction and analysis, and carryover was assessed by water blank injections between sample sets. Calibration curves for PFOA and PFOS were prepared over the range 0.01 to 150 ppb (µg L^-1^), showing good linearity (R^2^ > 0.990). The limit of detection (LoD) and limit of quantification (LoQ), defined at signal-to-noise ratios of at least 3 and 10, were 0.1 and 0.2 ng g^−1^, respectively, for both analytes. Recovery was estimated using ^13^C-labelled PFOA and PFOS internal standards and, when calculated within each species, was 95 ± 6% and 118 ± 4% in sunflower, 79 ± 10% and 126 ± 3% in maize, and 85 ± 5% and 111 ± 7% in hemp, for PFOA and PFOS, respectively. PFOS and PFOA concentrations in leaf, stem, and root tissues (C_l_, C_s_, and C_r_, respectively) were used to calculate key PFAS accumulation and translocation metrics. Bio Concentration Factor (BCF) was calculated as plant-to-soil concentration ratio relative to the nominal spiked concentration (500 µg kg^-1^ d.w. per compound; C_soil_). The tissue-specific BCFs of leaves, stems and roots (BCF_l_, BCF_s_, BCF_r_) were calculated as previously defined. The equations are reported as following:


$${\text{BCF}}_{\text{l}}=\;\frac{C_{l}}{\;C_{soil}}$$

$${\text{BCF}}_{\text{s}}=\;\frac{C_{\;s}}{\;C_{soil}}$$

$${\text{BCF}}_{\text{r}}=\;\frac{C_{r}}{\;\;C_{soil}}$$

For calculating the Bio Concentration Factor of the above ground biomass (BCF_ab_) and of total plant biomass (BCF_tot_), the latter including also roots, PFOA and PFOS concentrations in the aboveground biomass (C_ab_) and in the whole plant biomass (C_tot_) were used, respectively, in the following calculations:


$${\text{BCF}}_{\text{ab}}=\;\frac{\;C_{ab}}{\;C_{soil}}$$

$${\text{BCF}}_{\text{tot}}=\;\frac{\;C_{tot}}{C_{soil}}$$

BCF for aboveground biomass (BCF_ab_) was calculated as a biomass−weighted mean concentration of leaves and stems. Consequently, when stems have substantially lower PFAS concentrations than leaves, BCF_ab_ is lower than BCF_leaf_. A BCF > 1 indicates that the species is capable of bioaccumulating PFAS within its tissues, while higher BCF values indicate greater relative accumulation under the tested conditions.

Translocation Factor (TF), which represents the ratio between PFAS concentration in the aboveground biomass and that in the roots, was calculated to assess the mobility of PFOA and PFOS within the plant, as follows:


$$\text{TF}=\;\frac{\;C_{ab}}{C_{r}}$$

The amount of PFAS removed by the aboveground, and by total biomass of a single plant, known as the *Plant Burden*, was calculated by multiplying stem, leaf, and root d.w. by their respective PFAS concentrations.

Lastly, the Removal Efficiency (RE) for the aboveground and total plant biomass on a whole-pot basis (all plants) was calculated as the percentage of PFAS recovered in plant biomass, as following:


$${\text{RE}}_{\text{ab}}=\frac{Aboveground\;biomass\;burden\;\;\times \;n\;plants/pot}{m\;PFAS_{\;soil}}\;\times 100$$

$${\text{RE}}_{\text{tot}}=\frac{Total\;plant\;burden\;\;\times \;n\;plants/pot}{m\;PFAS_{\;soil}}\;\times 100$$

where “mPFAS_soil_” refers to the mass of PFOS, PFOA, or their sum spiked in each pot (1 mg for each PFAS, and 2 mg as total). Because soil PFAS concentrations were not measured at harvest, RE should be interpreted as the percentage of the initial spiked mass recovered in plant biomass rather than as a full pot-scale mass balance. Volatilization losses were expected to be negligible and substantial degradation was considered unlikely over the 32-day experiment, whereas sorption to the PET pot walls cannot be excluded and was not quantified directly.

These indices follow commonly applied approaches in PFAS phytoremediation research (Blaine et al., 2014; Huff et al., 2020; Mei et al., 2021; He et al., 2023; Nassazzi, 2023; Lesmeister et al., 2021). Tissue-level and biomass-based BCF metrics are mathematically distinct and were used as complementary indicators of PFAS accumulation and phytoextraction performance.

### 2.5 Statistical analysis

The data from all the assessed parameters were subjected to Analysis of Variance (ANOVA) using R ver. 3.4.4 (R Core Team, 2024) and RStudio ver. 2023.12.1 (Posit Software, PBC, 2009–2024) with key packages including car, emmeans, and pwr; significance was set at p ≤ 0.05. When ANOVA indicated significant treatment effects, mean separation was performed using Tukey-adjusted pairwise comparisons implemented with the emmeans package. Within each species, replication was balanced, with n=6 for hemp and n=3 for maize and sunflower, so the within-species *post hoc* comparisons are equivalent to the classical Tukey HSD procedure. Additional exploratory comparisons among species were conducted using models that included species as a factor. Because replication differed among species by design, cross-species pairwise contrasts were estimated using procedures appropriate for unequal sample sizes and were interpreted conservatively.

To quantify sensitivity, species−level minimum detectable effect sizes (MDES) were computed from the ANOVA residual variance using total d.w. plant biomass as the primary outcome (MDES *= d ×* SD_res_), where *d* is the Cohen’s *d* required for 80% power at α = 0.05 obtained with pwr.t.test. MDES values are reported in grams and as percent of the control (CTRL) mean to aid interpretation of non−significant contrasts (hemp: MDES ∼0.36 g, ∼23.2% of CTRL; maize: MDES ∼ 0.78 g, ∼15.9% of CTRL; sunflower: MDES ∼0.54 g, ∼18.4% of CTRL). For the total dry biomass model, normality of residuals was confirmed by the Shapiro-Wilk test (p ≥ 0.15) and homogeneity of variance by Levene’s test (p ≥ 0.35) across all species.

To explore multivariate treatment- and species-related patterns, principal component analysis (PCA) and factorial discriminant analysis (Multigroup Discriminant Analysis, MDA) with Wilks’ lambda and Pillai’s trace tests were conducted using MS Excel XLSTAT (Addinsoft, Paris, France). The data were standardized prior to analysis by subtracting the mean and dividing by the standard deviation within each variable. Because gas-exchange variables were measured only in hemp, their contribution to the multivariate space reflects within-hemp treatment variation; patterns associated with these traits were therefore interpreted as exploratory and not as cross-species physiological responses.

## 3 Results

### 3.1 Plant growth and physiological parameters

During the trial, the leaf chlorophyll SPAD index exhibited varying trends depending on the species and treatment (**Supplementary Figure 1**). In hemp, SPAD increased from a mean (all treatments) of 34 at 14 DAS to 53 at 32 DAS. In sunflower, the trend was less regular, with declines on 30 May (21 DAS) and 10 June (32 DAS).

Across most sampling dates, SPAD did not differ significantly among treatments in any species. In maize, however, treatment differences were significant at 28 DAS (p < 0.01), with higher SPAD values in HAs than in PFAS, and in CTRL than in PFAS (**Supplementary Figure 1**). SPAD values were numerically higher under PFAS treatment at the beginning of the measurement period, while the final samplings on 6 (28 DAS) and 10 June (32 DAS) generally showed a SPAD decrease compared to controls. SPAD values under HAs were slightly higher than the control across all three species, particularly in sunflower. Specifically, on 6 June (28 DAS), HAs resulted in increases of 3%, 5%, and 5% in hemp, maize, and sunflower, respectively, compared to the controls.

**Figure 1:**
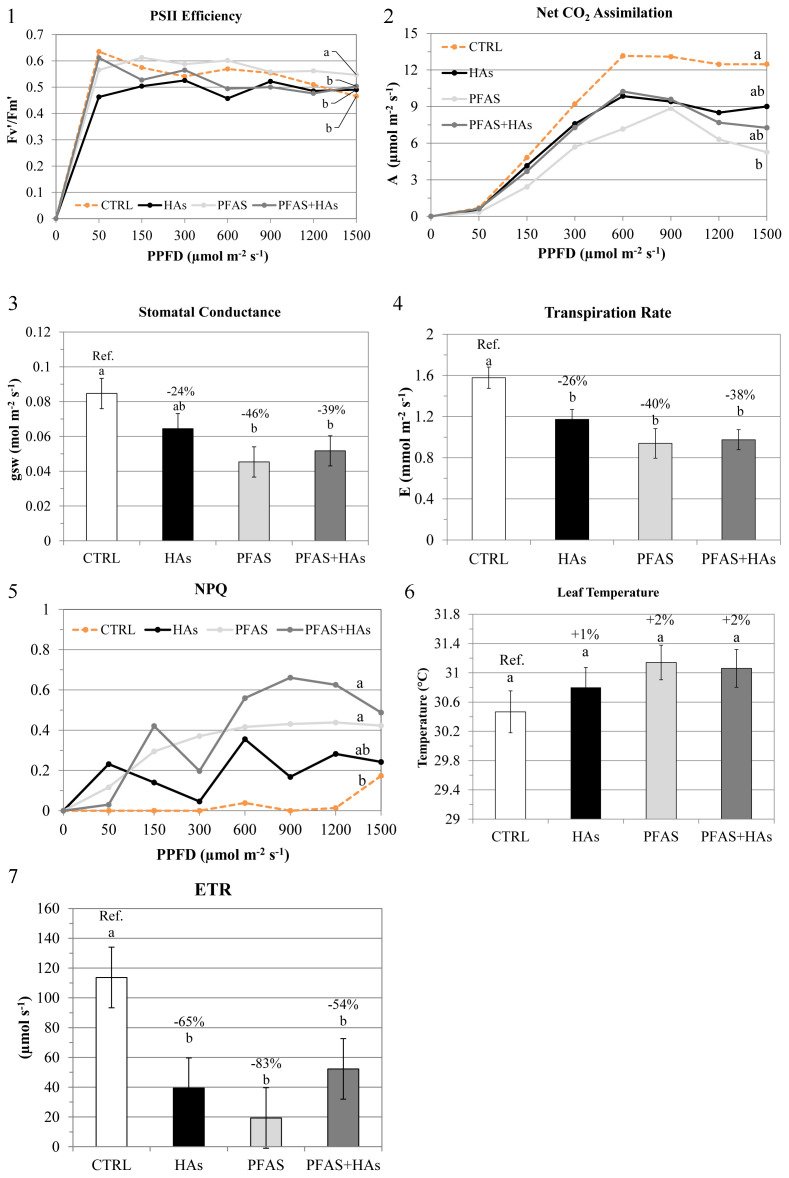
Photosynthetic parameters in hemp under CTRL (control), HAs (humic acids), PFAS (soil contamination), and PFAS+HAs (soil contamination + humic acids) treatments. Panel 1 shows PSII efficiency (Fv′/Fm′), panel 2 net CO_2_ assimilation (A), panel 3 stomatal conductance (gsw), panel 4 transpiration rate (E), panel 5 non-photochemical quenching (NPQ), panel 6 leaf temperature, and panel 7 electron transport rate (ETR) of PSII measured at 1,200 µmol m^−2^ s^−1^ PPFD. Panels 1, 2, and 5 present response curves across the PPFD (mean ± S.E.; n=6), whereas panels 3, 4, and 6 show values averaged across the eight applied PPFD levels (mean ± S.E.; n=48, i.e., 8 PPFD levels × 6 replicates). Panel 7 reports mean ± S.E. values (n=6), with percentages indicating variation relative to the control (Ref.). Letters indicate significant differences among treatments (across the whole ramp for Fv′/Fm′, A and NPQ) according to Tukey’s test (p ≤ 0.05).

Regarding the photosynthetic parameters, at 26 DAS, PFAS-treated hemp showed significantly higher PSII operating efficiency (Fv′/Fm′) than the control across the PPFD ramp. In contrast, the HAs and PFAS+HAs treatments did not differ significantly from the control or from each other (**Figure 1**, panel 1). PFAS treatment also significantly reduced net CO_2_ assimilation rate across the PPFD range, with values lower than the control over most of the irradiance sequence (**Figure 1**, panel 2).

Compared to the control, PFAS also significantly increased NPQ across the PPFD range, irrespective of humic acid application (**Figure 1**, panel 5). Simultaneously, electron transport rate (ETR) at 1,200 µmol m^-2^ s^-1^PPFD was significantly reduced in all treatments relative to the control (**Figure 1**, panel 7). The largest decrease was observed under PFAS treatment (−83%), followed by HAs (−65%) and PFAS+HAs (−54%). Despite the higher PSII operating efficiency observed under PFAS, ETR at near-saturating irradiance was lower than in the control.

Regarding stomatal conductance (gsw), significant differences were also observed among treatments in hemp. Specifically, PFAS and PFAS+HAs resulted in significantly lower gsw values, with reductions of 46% and 39%, respectively, compared to the control. Leaf transpiration rate was significantly higher in the control than all other treatments, with PFAS treatments showing the largest reduction (−40% and −38% vs. CTRL). A slight increase in leaf temperature was also observed under PFAS treatments (approximately +2%; 31.14 °C and 31.06 °C under PFAS and PFAS+HAs, respectively, vs. 30.47 °C in the control), but differences among treatments were not statistically significant (p > 0.05).

No visible differences were observed in plant growth or leaf greenness among the treatments in any of the three species during the trial and at harvest (**Supplementary Figure 2**). Plant height trends were generally uniform, with no notable variations among treatments (**Supplementary Figure 3**). At the final sampling on 10 June (32 DAS), plants treated with humic acids (HAs) showed an increase in height compared to the control: +8%, +5%, and +9% in hemp, maize, and sunflower, respectively. On this date (32 DAS), a significant difference was observed in hemp among treatments, with HAs being higher than the PFAS treatment (p ≤ 0.05): the average height in the HAs treatment was 449 mm, which was 35 mm higher than the control (414 mm).

### 3.2 Biomass dry weight assessment

**Figure 2:**
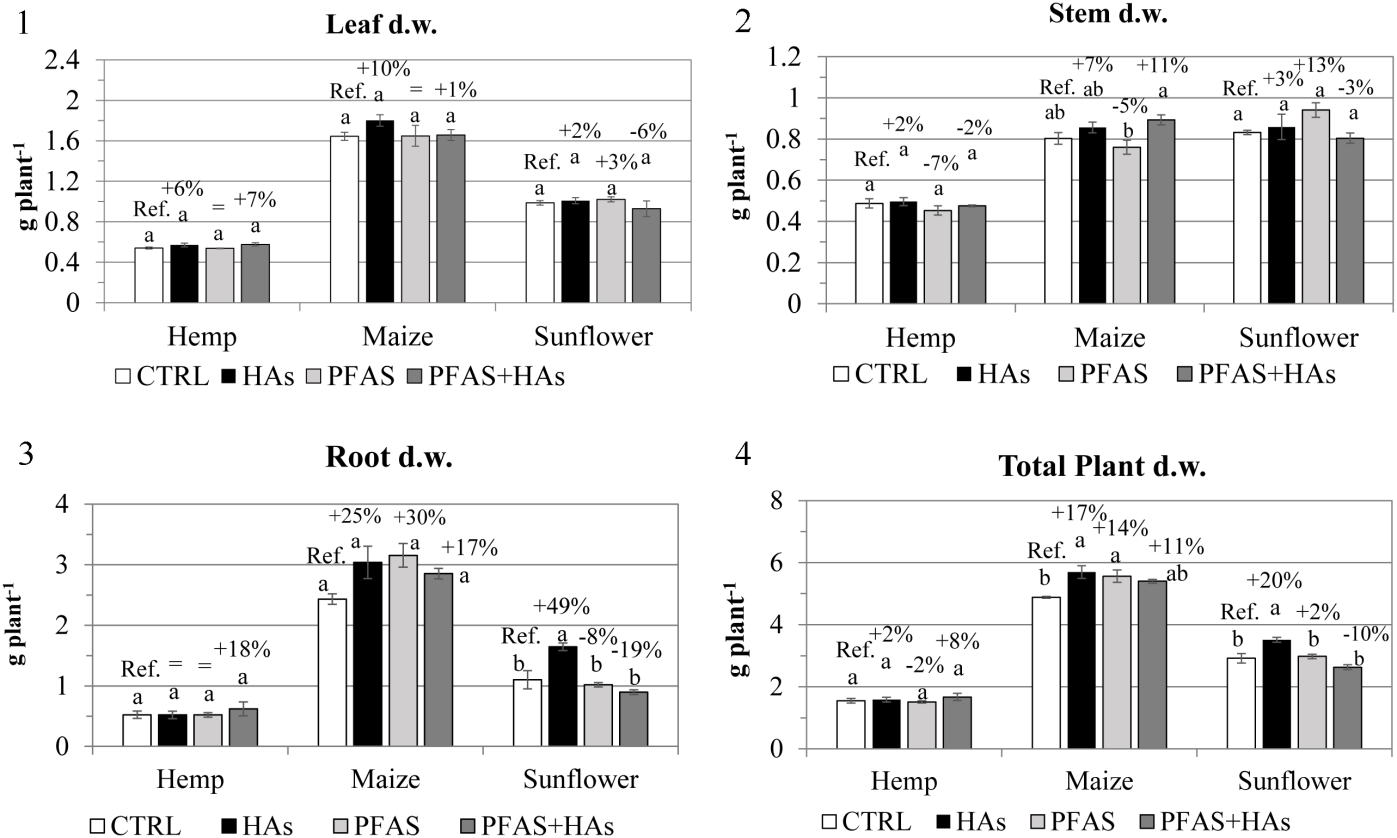
Leaf, stem, root, and total plant biomass dry weight (d.w. plant^−1^) in hemp (CS; mean ± S.E.; n=6), maize (ZM; mean ± S.E.; n=3), and sunflower (HA; mean ± S.E.; n=3) under CTRL (control), HAs (humic acids), PFAS (soil contamination), and PFAS+HAs (soil contamination + humic acids) treatments, shown in Panels 1, 2, 3, and 4, respectively. Percentages: variation relative to control (Ref.). Letters: statistical differences among treatments within the same species (Tukey’s test, p ≤ 0.05). Note that the Y-axis scale is different among panels.

Measurements taken at harvest (32 DAS) revealed few statistically significant differences in dry weight among treatments and the control for leaf, stem, and root compartments across all three species examined (**Figure 2**). Descriptive percentage differences were observed among treatments, but most were not statistically significant. In maize, stem biomass showed a 5% decrease under the PFAS treatment compared with the control, while an increase was recorded under PFAS+HAs (+11%). In hemp, stem biomass was 7% lower under PFAS and 2% lower under PFAS+HAs than in the control. In sunflower, stem biomass was 13% higher under PFAS and 3% lower under PFAS+HAs than in the control, although these differences were not significant.

Leaf biomass remained generally stable or slightly increased under PFAS across all species and treatments, with the exception of sunflower under PFAS+HAs (–6%, n.s.). At the organ level, the only statistically significant effect was observed in sunflower roots, where HAs alone increased root biomass by 49% compared with the control. Hemp root biomass showed only minor variations across treatments, with a variation (+18%) under PFAS+HAs only, and no significant differences among treatments.

At the whole plant level, total biomass production did not differ significantly between PFAS-treated plants and the control at the spiked concentrations. Relative to controls, total dry weight showed a slight reduction in hemp (−2%) and a slight increase in sunflower (+2%), both non-significant. By contrast, total dry weight was significantly increased in the PFAS treatment of maize (+14%, p ≤ 0.05).

The PFAS+HAs treatment showed similar results, with total plant biomass 8% and 11% higher in hemp and maize, respectively, and 10% lower in sunflower; however, these differences were not significant. Under HAs alone, total plant dry weight was higher than the control in all three species, with statistically significant increases observed in maize (+17%) and sunflower (+20%) compared to the control, whereas the increase in hemp was minimal (+2% n.s.). Under PFAS+HAs, total biomass values were lower than under HAs alone and were not significantly different from the control. In hemp, no significant differences in total plant dry weight were observed among treatments, while in maize, total biomass was significantly higher under PFAS and HAs alone than in the control, whereas in sunflower a significant increase was detected only for HAs alone.

### 3.3 PFAS concentration in plant biomass

**Figure 3:**
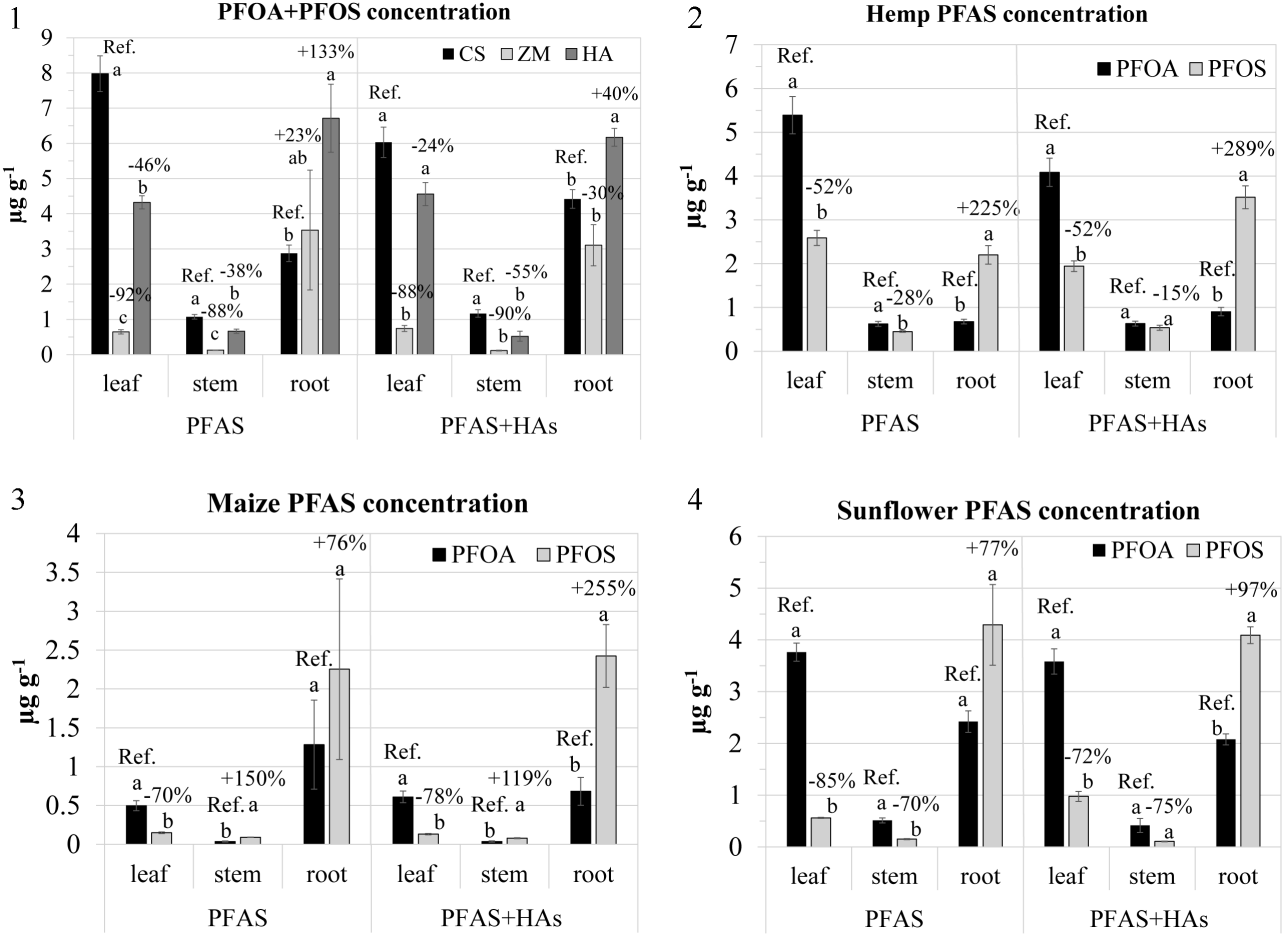
PFOA+PFOS concentration in leaves, stems, and roots of hemp (CS), maize (ZM), and sunflower (HA) under PFAS (soil contamination), and PFAS+HAs (soil contamination + humic acids) treatments. Panel 1 reports PFOA+PFOS concentration in hemp (CS; mean ± S.E.; n=6), maize (ZM) and sunflower (HA) (mean ± S.E.; n=3). Panels 2, 3, and 4 show PFOA and PFOS concentrations in leaves, stems, and roots of hemp (mean ± S.E.; n=6), maize and sunflower (mean ± S.E.; n=3), under PFAS and PFAS+HAs treatments. Percentages: variations relative to reference (Ref.). Letters: statistical comparison within the factor under study, namely species in Panel 1 and PFOA vs. PFOS in Panels 2, 3, and 4, according to Tukey’s test (p ≤ 0.05).

LC-MS/MS analysis revealed clear differences in total PFOA+PFOS concentration among plant compartments under PFAS and PFAS+HAs treatments (**Figure 3**, panel 1). The highest PFOA+PFOS concentration (7.98 µg g^-1^ d.w.) was measured in hemp leaves under the PFAS treatment. Descriptively, hemp showed the highest concentrations in aboveground organs (leaves and stems) under both PFAS and PFAS+HAs treatments, followed by sunflower and maize.

Under PFAS treatment, leaf PFOA+PFOS concentrations were significantly lower in sunflower (−46%) and maize (−92%) compared to hemp. In contrast, sunflower exhibited the highest root PFOA+PFOS concentrations (6.71 and 6.17 µg g^-1^ d.w. in PFAS and PFAS+HAs, respectively) (**Figure 3**, panel 1). These values were significantly higher than those in hemp roots (2.88 and 4.42 µg g^-1^ d.w., in PFAS and PFAS+HAs, respectively, p ≤ 0.05).

In hemp, leaf PFOS concentration was significantly lower than that of PFOA, showing a 52% reduction under both PFAS and PFAS+HAs treatments (**Figure 3**, panel 2). The difference between the two contaminants was less pronounced in stems, whereas roots exhibited an opposite trend, with PFOS being significantly more accumulated than PFOA. Specifically, root PFOS accumulated to 2.20 µg g^-1^ d.w., which was 225% higher than root PFOA under PFAS treatment. Similarly, in sunflower and maize leaves, PFOA concentration was significantly higher than PFOS, and PFOS was more concentrated in the roots, regardless of soil HAs application (**Figure 3**, panels 3 and 4).

In hemp, PFAS concentration in stems was significantly lower than that in leaves for both PFOS (5.8 times lower) and PFOA (8.6 times lower) under PFAS treatment. In this plant species, leaf PFOA and PFOS concentrations reached 5.39 and 2.59 µg g^-1^ d.w., respectively, while stem concentrations were notably lower (0.62 and 0.45 µg g^-1^ d.w.) (**Figure 3**, panel 2).

Regarding the concentration of contaminant in the whole plant biomass, soil application of HAs did not significantly affect the concentrations of PFOA, PFOS, or their sum in any of the species (p > 0.05) (**Supplementary Figure 4**, panel 1). However, directional trends were observed: in hemp, PFOA concentration tended to be 14% lower under PFAS+HAs, while PFOS tended to be 12% higher. In maize and sunflower, directional changes followed similar patterns of divergence between the two compounds.

When averaged across all three species (main effect), PFOA+PFOS concentrations in leaves and roots were significantly higher than those measured in stems under both PFAS and PFAS+HAs treatments (**Supplementary Figure 4**, panel 2). At the same time, root concentrations increased from 4 µg g^-1^ under PFAS to 4.53 µg g^-1^ under PFAS+HAs treatment, leaf concentration slightly decreased, and concentration in the stem remained stable (**Supplementary Figure 4**, panel 2).

### 3.4 BCF, TF and PFAS plant burden

**Figure 4:**
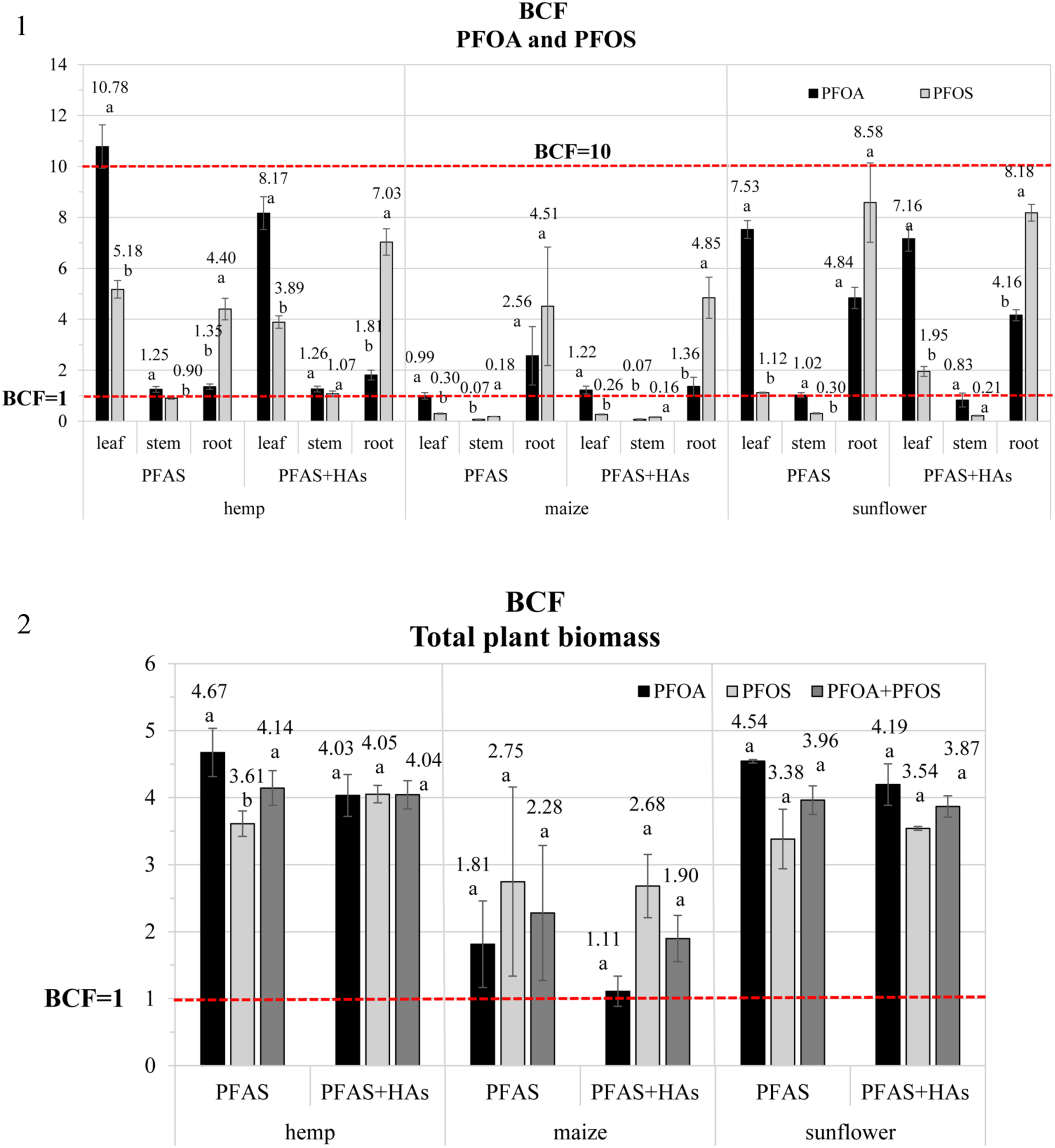
Bioconcentration factor (BCF) of PFOA and PFOS in leaves, stems, and roots, and in total plant biomass of hemp (CS), maize (ZM), and sunflower (HA) under PFAS (soil contamination), and PFAS+HAs (soil contamination + humic acids) treatments. Panel 1 shows tissue-level BCF values for PFOA and PFOS in leaves, stems, and roots (BCF_l_, BCF_s_, BCF_r_) of hemp (mean ± S.E.; n=6), maize, and sunflower (mean ± S.E.; n=3). Values were calculated as the ratio between the PFAS concentration in each tissue and the initial soil concentration. Panel 2 shows total plant BCF (BCF_tot_) calculated from whole-plant PFOA or PFOS concentrations. Values are reported above bars. Letters: statistical comparison among pollutants PFOA and PFOS (and PFOA+PFOS in panel 2) (Tukey’s test, p ≤ 0.05). Red horizontal dashed lines indicate reference values at BCF = 1 and BCF = 10.

The bioconcentration factor (BCF) varied significantly among species and plant organs. When BCF was calculated for leaf tissue only, PFOA consistently exceeded PFOS in all the three investigated plant species (**Figure 4**, panel 1).

Hemp showed the highest leaf BCF values reaching 10.78 for PFOA and 5.18 for PFOS under the PFAS treatment. Sunflower also showed relatively high BCF values in both leaves and roots (>7), whereas stems of both hemp and sunflower displayed a BCF below 1.26. Maize exhibited a contrasting pattern: BCF rarely exceeded 1 in leaves and stems in both treatments (PFAS and PFAS+HAs) for both contaminants. In maize, the highest BCF values were observed in roots, particularly for PFOS, with BCF values of 4.51 and 4.85 under PFAS and PFAS+HAs treatments, respectively.

Referring to the aboveground plant biomass (leaves+stem; **Supplementary Figure 5**, panel 1), BCF_ab_ in maize remained below 1 for both pollutants, whereas hemp and sunflower showed higher phytoextraction and accumulation capacities in aboveground biomass, particularly for PFOA, with BCF_ab_ values of 6.42 (hemp) and 4.41 (sunflower), under PFAS treatment.

The difference between maize and the other species were smaller when considering the total plant biomass, that is BCF_tot_ as opposed to BCF_ab_ (**Figure 4**, panel 2). Maize showed BCF_tot_ values ranging from 1.11 to 2.75, with higher whole-plant values than aboveground values. However, hemp and sunflower showed the highest BCF_tot_, with values for PFOA two-to-three times higher than in maize. Indeed, under PFAS treatment, BCF_tot_ values for PFOA were 4.67 for hemp and 4.54 for sunflower compared to 1.81 recorded in maize. For PFOS, the differences were narrower, with BCF_tot_ values of 3.61 for hemp, 3.38 for sunflower, and 2.75 for maize, under PFAS treatment. Under PFAS+HAs, BCF_tot_ values were numerically lower than under PFAS in several comparisons.

**Figure 5:**
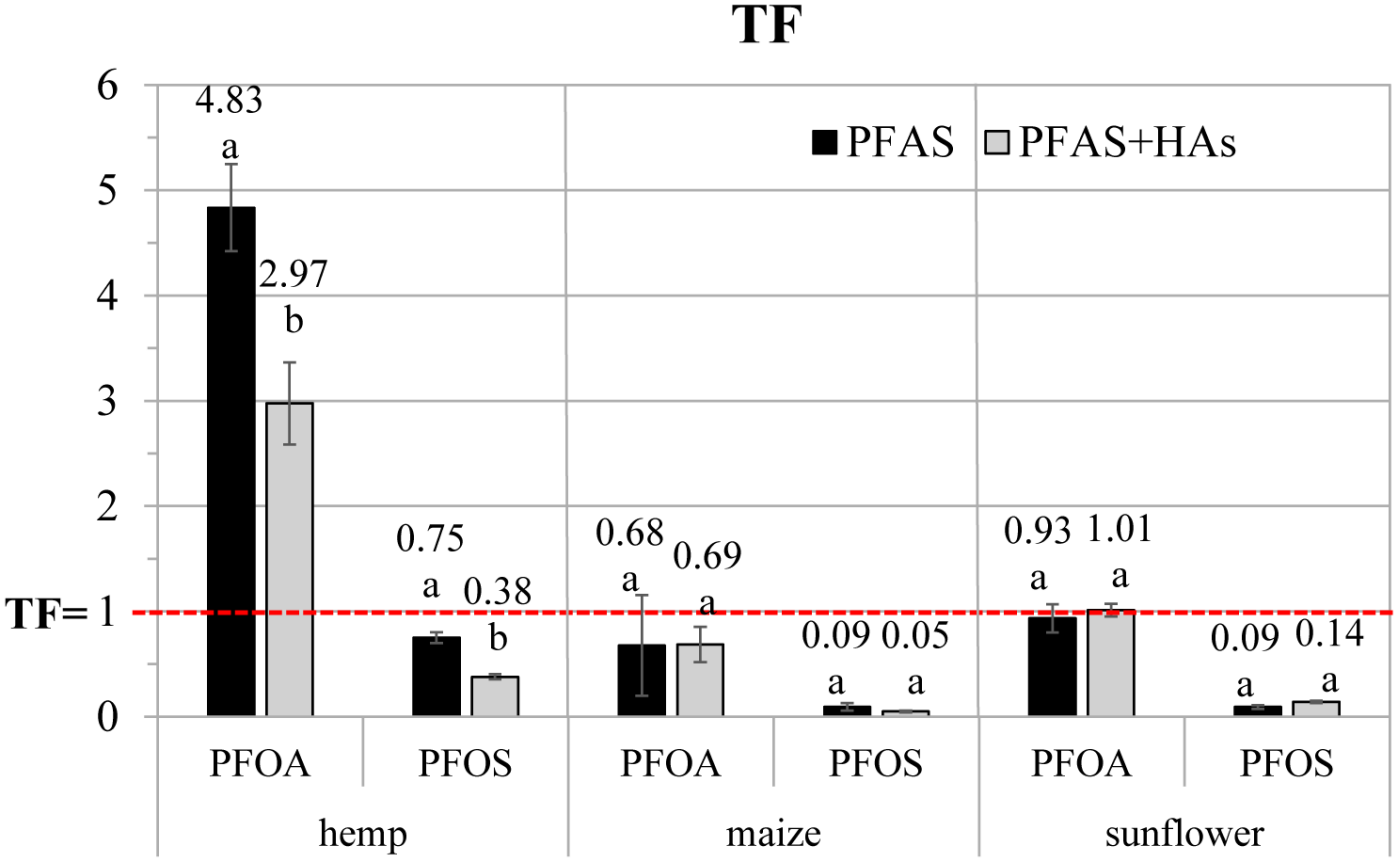
Translocation factor (TF) of PFOA and PFOS in hemp (mean ± S.E.; n=6), maize and sunflower plants (mean ± S.E.; n=3), under PFAS (soil contamination), and PFAS+HAs (soil contamination + humic acids) treatments. TF values are reported above bars. Letters: statistical comparison between PFAS and PFAS+HAs treatments (Tukey’s test, p ≤ 0.05). The horizontal dashed red line at TF = 1 indicates the reference point, and TF > 1 marks pollutant concentration in shoots exceeding that in roots.

The Translocation Factor (TF) differed among species (**Figure 5**). Hemp showed the highest TF values, particularly for PFOA, with values of 4.83 under PFAS and 2.97 under PFAS+HAs. In maize and sunflower, TF values remained much lower overall, especially for PFOS, the latter ranging from 0.05 to 0.14. A significant difference between PFAS and PFAS+HAs was observed only in hemp for both PFOA and PFOS, with a TF reduction under PFAS+HAs, whereas no significant treatment effect was detected in maize or sunflower.

**Figure 6:**
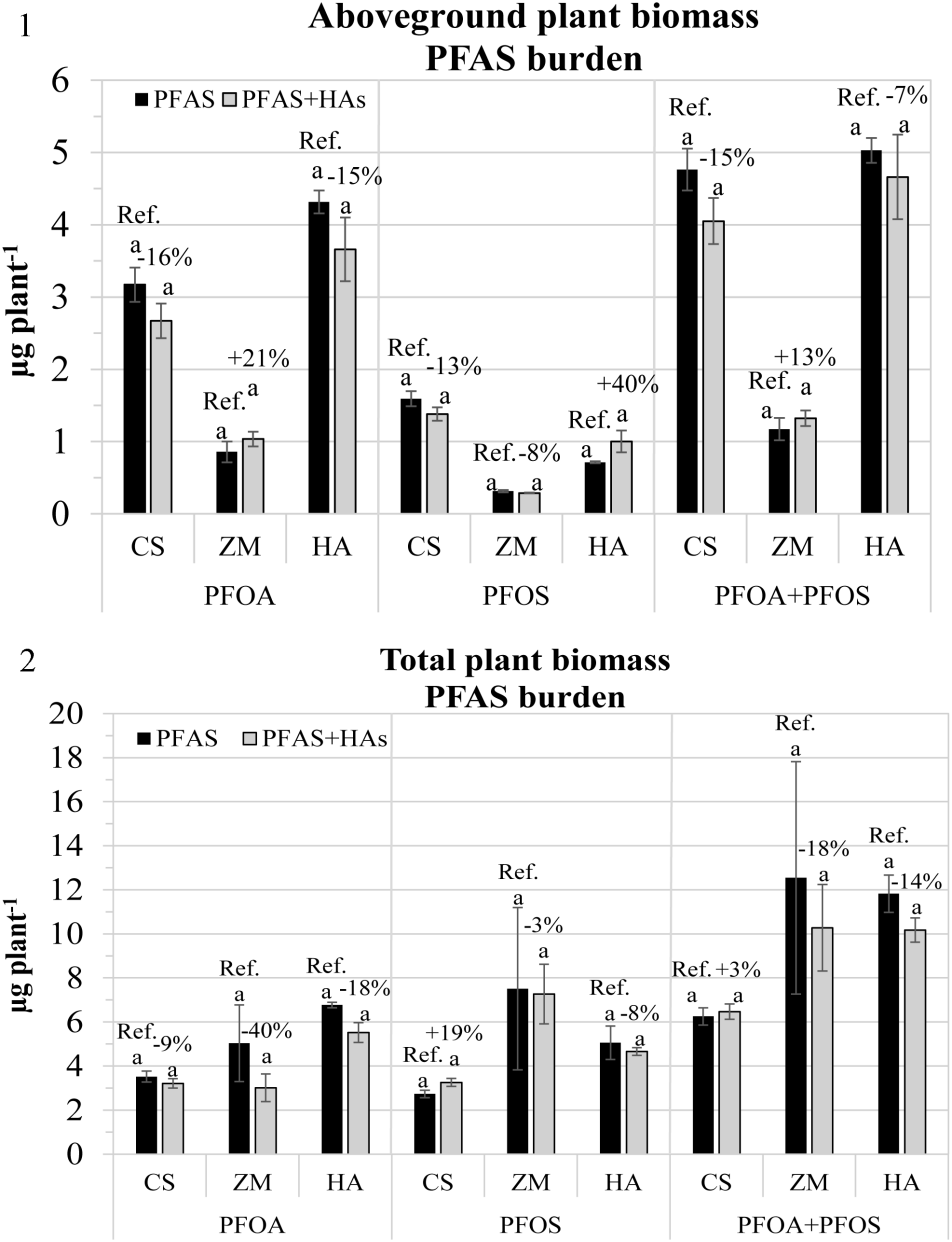
PFAS burden in aboveground and total plant biomass of hemp (CS), maize (ZM), and sunflower (HA) under PFAS (soil contamination), and PFAS+HAs (soil contamination + humic acids) treatments. Panel 1 shows the aboveground plant biomass burden of PFOA, PFOS, and PFOA+PFOS in hemp (mean ± S.E.; n=6), maize, and sunflower (mean ± S.E.; n=3). Panel 2 shows the total plant biomass burden of PFOA, PFOS, and PFOA+PFOS in hemp (mean ± S.E.; n=6), maize, and sunflower (mean ± S.E.; n=3). Percentages: variation vs. PFAS treatment (Ref.). Letters: statistical comparison between PFAS and PFAS+HAs treatments within the same plant species and pollutant (Tukey’s test, p ≤ 0.05).

The derived plant burden, that is, the total amount of PFAS absorbed by an individual plant, both in the aboveground biomass (**Figure 6**, panel 1) and in total plant biomass (**Figure 6**, panel 2), showed significant differences among species and pollutant type. No statistically significant differences were observed between PFAS and PFAS+HAs treatments. Hemp and sunflower showed a higher PFAS removal (PFOA+PFOS) with their aboveground biomass under the PFAS treatment (without humic acids), whereas maize showed slightly higher value under soil application with HAs (+13% compared to the treatment with only PFAS, n.s.).

In terms of PFAS removal by the aboveground biomass, under PFAS treatment, sunflower accumulated 4.31 µg plant^-1^ of PFOA compared to 3.17 µg plant^-1^ of hemp. Hemp nevertheless showed higher aboveground biomass PFOS removal, with 1.59 and 1.38 µg plant^-1^, compared to sunflower’s 0.71 and 1.00 µg plant^-1^ under PFAS and PFAS+HAs treatments, respectively. Combined PFOA+PFOS removals were highest in the aboveground biomass of hemp and sunflower, with values ranging from 4.05 to 5.02 µg plant^-1^, significantly higher than in maize (1.17 and 1.32 µg plant^-1^ under PFAS and PFAS+HAs treatments).

When considering total plant biomass PFAS burden, including root contribution, hemp exhibited the lowest removal for both contaminants. In maize, total removal capacity, particularly for PFOS was 7.51 and 7.27 µg plant^-1^ under PFAS and PFAS+HAs treatments, respectively. Sunflower showed the highest removal of PFOA (6.77 and 5.52 µg plant^-1^ under PFAS and PFAS+HAs treatments, respectively).

For combined PFOA+PFOS, total plant burden was significantly higher in maize and sunflower than in hemp, with values ranging from 10.18 to 12.55 µg plant^-1^ in maize and sunflower, and from 6.25 to 6.47 µg plant^-1^ in hemp under PFAS and PFAS+HAs treatments. These values are reported on a per-plant basis.

Indeed, the removal efficiency by the aboveground plant biomass of the three species in a pot (considering plant number) was as follows: hemp > sunflower > maize (**Supplementary Figure 6**, panel 1) for both individual pollutants (PFOA and PFOS). For the sum PFOA+PFOS, aboveground removal efficiency followed the same species order. The highest aboveground biomass removal efficiency was observed for PFAS-treated hemp (without HAs) and for PFOA, achieving a Removal Efficiency of 1.59%. Under the same treatment and pollutant, maize and sunflower showed significantly lower efficiencies (−84% and −18%) than hemp, respectively. Similarly, hemp also exhibited the highest removal efficiency of PFOS through the aboveground biomass, although it was approximately half that of PFOA (0.8%). Under PFAS+HAs, aboveground removal efficiency of PFOA+PFOS was numerically lower (n.s.) than under PFAS in hemp and sunflower, but not in maize.

Considering the whole plant biomass (aboveground + roots), maize showed the highest overall plant removal efficiency for PFOS. Although it was not significantly higher than the other two species, with values slightly exceeding 2% (2.25% and 2.18% under PFAS and PFAS+HAs treatments, respectively) (**Supplementary Figure 6**, panel 2). In contrast, hemp and sunflower exhibited lower efficiencies, around 1.5% for PFOS. For PFOA, removal efficiency ranged from 1.5% to 2% among species, with no significant differences, except for a statistically significant decrease in maize under the PFAS+HAs treatment.

Lastly, no significant differences were observed among species regarding the overall removal efficiency of PFOA+PFOS, with values ranging between 1.53% and 1.88%.

### 3.5 PCA and MDA

**Figure 7:**
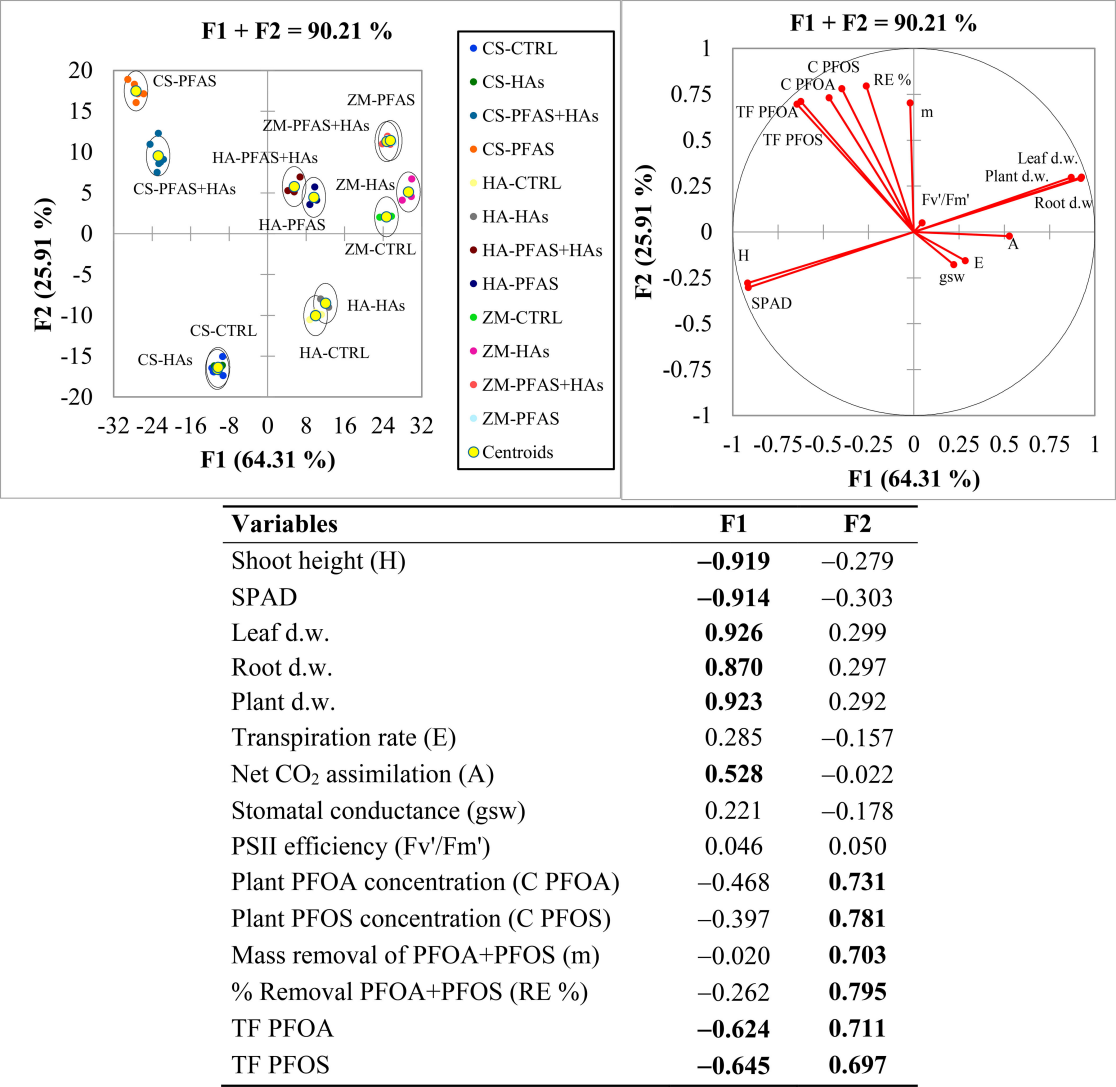
Multigroup discriminant analysis (MDA; top left) and principal component analysis (PCA; top right) of growth, photosynthetic parameters and PFAS uptake in hemp (CS), maize (ZM) and sunflower (HA) under CTRL, HAs, PFAS and PFAS+HAs treatments. The isodensity confidence circles contain 75% of variability. In the bottom table, the highly informative variables loadings greater than |0.5|, are highlighted in bold within the synthetic variables F1 and F2. Note that photosynthetic variables refer to hemp only.

Principal Component Analysis (PCA) identified two main synthetic variables, F1 and F2, which together explained 90.21% of the total variance (64.31% by F1 and 25.91% by F2) (**Figure 7**). The most influential variables (loadings >|0.5|) were the biomass of all plant parts, leaf chlorophyll content, and CO_2_ net assimilation for F1, while PFAS concentrations, translocation, and removal rates were associated mainly with F2. Because gas-exchange variables were measured only in hemp, their loadings in the PCA reflect within-hemp treatment variation and should not be interpreted as evidence of cross-species physiological separation.

Based on the variable loadings and vector orientations in the PCA biplot, a positive correlation between SPAD and plant height, and between stomatal conductance and leaf transpiration was observed. In contrast, the PCA biplot showed an opposite orientation between photosynthetic variables (for example, CO_2_ assimilation, stomatal conductance, and transpiration) and PFAS accumulation and root-to-shoot translocation variables. This pattern was descriptive only and was not followed by a formal correlation analysis.

Based on the centroid position and cluster separation, Multivariate Discriminant Analysis (MDA) showed separation among the three species. In the ordination space, hemp was positioned with higher values of tissue PFAS concentration and translocation-related variables, as indicated by elevated concentrations of PFOA and PFOS as well as high translocation factor (TF) values (**Figure 7**, left). Maize and sunflower were more closely associated with variables related to biomass production and root-associated PFAS accumulation. This species-level separation should be interpreted cautiously, as it reflects a combination of unequal replication among species, and the inclusion of gas-exchange variables measured only in hemp.

Across species, SPAD values, plant height, or total plant biomass were generally comparable across treatments and regardless of humic acid application. In hemp PFAS treatment was associated with lower net CO_2_ assimilation, stomatal conductance, and transpiration than the control.

## 4 Discussion

### 4.1 Morphological integrity and photosynthetic stress responses under PFAS exposure

No macroscopic phytotoxicity was observed in any of the three species cultivated in soil spiked with 500 µg kg^-1^ of both PFOA and PFOS, and total dry weight was not statistically reduced. This maintenance of morphological integrity suggests short-term PFAS tolerance at the whole-plant level, although it does not exclude subcellular or physiological stress. For this reason, maintained biomass should not be equated with true physiological tolerance. In hemp, Luche et al. (2026a) showed that *in-vitro*-grown plantlets exposed to 1 mg L^-1^ PFOA or PFOS exhibited significant DNA fragmentation and oxidative DNA damage (8-oxo-deoxyguanosine accumulation) despite unchanged or even increased shoot biomass and chlorophyll content. These authors proposed that the observed growth stimulation under PFAS exposure may reflect an over-compensatory response triggered by cellular homeostasis disruption rather than a truly beneficial effect. A similar interpretation may apply to the significant 14% increase in total biomass observed in maize under PFAS exposure in the present study, although this result should be interpreted cautiously given the early vegetative stage examined and the limited replication for this species. Such apparent resilience is nonetheless consistent with documented PFAS interactions with cellular membranes, including interference with ion transport and metabolic processes in plants (Liu et al., 2023; Karamat et al., 2024).

The maintenance of total biomass at sub-lethal concentrations is consistent with previous studies. Gobelius et al. (2017) reported minimal visible phytotoxicity in grasses such as common reed (*Phragmites australis*) and Kentucky bluegrass (*Poa pratensis*), exposed to up to 34 µg L^-1^ PFAS in the groundwater near firefighting training facilities. Although the concentrations used in that study was substantially lower than 1,000 µg kg^-1^ applied in our trial, a similar absence of visible macroscopic symptoms was observed. This may be partly explained by the capacity of plant tissues to temporarily sequester these pollutants within vacuoles or cell walls, effectively isolating them from essential cellular physiological pathways (Tang et al., 2022). Plants may also activate detoxification mechanisms, including increased production of antioxidant enzymes to counteract oxidative stress induced by PFAS exposure (Li et al., 2022). However, recent evidence indicates that PFAS accumulation can also inhibit antioxidant enzyme activity, at least in hemp plantlets (Luche et al., 2026a), which suggests that stress mitigation is likely species- and context-dependent.

In hemp PFAS treatment produced a paradoxical photosynthetic response: PSII operating efficiency (Fv’/Fm’) increased, while stomatal conductance (gsw) and transpiration (E) and net CO_2_ assimilation (A) declined significantly across a large PPFD ramp. This pattern is not consistent with typical PSII inhibition, which lowers Fv’/Fm’ (Mallick and Mohn, 2003), but instead suggests that PSII photochemistry remained relatively functional while downstream carbon fixation was constrained, pointing to a bottleneck beyond PSII (Atkin and Macherel, 2009; Flexas et al., 2004). This pattern has not been previously described for PFAS exposure in soil-grown hemp, highlighting a gap in understanding how PFAS affect PSII operating efficiency. It may represent a short-term regulatory photoprotective regulatory response specific to the growth conditions and exposure concentrations used here. Partial parallels exist in the literature. Battisti et al. (2023) observed similar stomatal closure in PFAS-exposed willow grown in hydroponics, whereas Sharma et al. (2020) reported increased assimilation and stomatal conductance in willow under different PFAS exposure scenario. These contrasting findings suggest that PFAS effects on gas exchange are species-specific, influenced by exposure conditions, and reflect divergent hydraulic and stomatal adjustments. Potential sources of this variability include PFAS concentration, composition and their physicochemical properties, exposure system and growth matrix, plant species and developmental stage, all of which can influence PFAS bioavailability, translocation, plant water relations, and stomatal sensitivity (Sharma et al., 2020; Blaine et al., 2014; Costello and Lee, 2020).

From a mechanistic perspective, the simultaneous increase in Fv′/Fm′ and decrease in net CO_2_ assimilation is consistent with a classic stomatal limitation scenario (Farquhar and Sharkey, 1982). PFAS-induced stomatal closure, evidenced by the 46% reduction in gsw, would restrict CO_2_ supply to the mesophyll and reduce Calvin cycle demand the for photochemical energy. This shift in the balance between energy absorption and assimilation would produce an apparent increase in PSII operating efficiency (Fv′/Fm′) and promotes NPQ as a photoprotective response. The observed increase in NPQ supports this interpretation (**Figure 1**, panel 5). However, this explanation remains a working hypothesis without direct measurements of mesophyll conductance or intercellular−to−ambient CO_2_ ratio. The biochemical limitation of photosynthesis, such as reduced Rubisco carboxylation capacity, therefore cannot be excluded. Both diffusional and biochemical constraints may have contributed to the decline in CO_2_ assimilation (Farquhar et al., 1980; Flexas et al., 2004, 2008).

In general, stressors that directly impair PSII, also lower the Fv’/Fm’ (Mallick and Mohn, 2003). The elevated Fv’/Fm’ observed here therefore does not signify improved PSII function, but reflects a compensatory adjustment. Such an adjustment may involve altered antenna configuration, pigment composition, or increased energy dissipation via NPQ, temporarily maintaining PSII efficiency under low-to-moderate irradiance despite underlying stress. The slight increase in leaf temperature under PFAS treatment (~ +2%) is consistent with greater dissipation of excess energy as heat and reduced stomatal conductance. Overall, PFAS interference appears localized downstream of PSII, likely via diffusive CO_2_ limitations or impaired biochemical sinks (e.g., Rubisco activity). These constraints would be expected to reduce electron use by carbon fixation and thereby contribute to lower ETR. Under such conditions, stronger NPQ and related photoprotective down-regulation could alter fluorescence relationships in a way that increases the calculated Fv’/Fm’ ratio. Accordingly, the elevated Fv’/Fm’ ratio is best interpreted as a sign of photoprotective adjustment under downstream metabolic limitation rather than as improved photosynthetic performance (Lichtenthaler et al., 2013; Wilkinson et al., 2017). In particular, combining dark-adapted Fv/Fm measurements with light-adapted fluorescence would help determine whether the observed response reflects persistent PSII damage or a reversible photoprotective adjustment associated with altered energy partitioning.

Recent molecular evidence could support this interpretation; proteomic analysis by Luche et al. (2026a) showed that PFOA exposure in hemp upregulated proteins involved in chlorophyll biosynthesis, whereas PFOS downregulated gluconeogenesis enzymes, specifically phosphoenolpyruvate carboxykinase and fructose-1,6-bisphosphatase. This differential response is consistent with our observation that light-harvesting was maintained, whereas carbon fixation was reduced. A further mechanistic hypothesis is that PFAS may interact with thylakoid membranes and thereby alter the local micro-environment of PSII, including the oxygen-evolving complex (OEC), which depends on a tightly regulated ionic environment for efficient water oxidation (Wincencjusz et al., 1999). Although speculative and not directly supported by the present data, this interpretation is consistent with the combined gas−exchange and fluorescence patterns observed here and therefore warrants targeted investigation in future studies.

The decline in carbon assimilation was most likely driven by stomatal closure limiting CO_2_ availability for its fixation in the Calvin cycle, particularly in a C3 species such as hemp (Flexas et al., 2004). However, diffusive constraints regarding CO_2_ alone do not exclude concurrent metabolic inhibition of carboxylation. Notably, despite markedly reduced CO_2_ assimilation at 26 DAS, total biomass at harvest (32 DAS) did not decline compared to the control. This maintenance of growth should be interpreted strictly within the 32-day early vegetative stage examined and does not warrant extrapolation to full-cycle agronomic performance. One possible interpretation is that the assimilation rate may have remained above the plant compensation point over the short term, and/or the plants may have reallocated resources to essential structures or curbed respiratory carbon losses to maintain biomass production. Such adjustments would improve short-term carbon use efficiency and help explain how growth was sustained despite a physiological stress (Flexas et al., 2006; Hartmann et al., 2020). Because respiratory measurements were not conducted, the compensation-point hypothesis remains speculative. As gas-exchange measurements were limited to hemp, these mechanistic interpretations cannot be extrapolated directly to sunflower, and particularly to maize, which differ in photosynthetic pathway and may respond differently to PFAS exposure.

Accordingly, retrospective sensitivity analysis of total dry biomass indicates that the n=3 design used for maize and sunflower was able to detect moderate biomass differences under the observed residual variance, although subtle effects, and especially effects in endpoints other than total biomass, may not have been detected. Visual observations (**Supplementary Figure 2**) confirmed the absence of shoot phytotoxicity in all three species, and the lack of significant differences in total dry weight indicates that the PFOA+PFOS mixture was not overly phytotoxic at the applied concentration. The absence of significant differences in leaf, stem, and root dry weight does not imply that PFAS exposure had no effect. Rather, it indicates that the selected concentration of PFAS (1,000 µg kg^–1^ as PFOA+PFOS) was below the threshold for detectable growth inhibition under the tested conditions. This concentration was intentionally chosen to characterize PFAS accumulation and sub−lethal physiological responses, rather than to determine a phytotoxic threshold or IC50 (half−maximal inhibitory concentration). This outcome is consistent with a recent meta-analysis of 776 observations across 68 studies, which showed that PFAS phytotoxicity on plant biomass is strongly concentration-dependent, with significant reductions primarily evident at supra-environmental exposure levels (Luche et al., 2026b). Previous studies confirm that PFAS can accumulate in plants at environmentally relevant concentrations without causing visible growth inhibition, while still triggering physiological, metabolic and antioxidant adjustments (Li et al., 2020; Pietrini et al., 2024).

This apparent absence of macroscopic phytotoxicity is encouraging for potential phytoremediation use of these species, although it must be viewed with caution.

### 4.2 Species-specific PFAS accumulation and removal patterns

PFOA+PFOS concentrations in total plant biomass were higher in hemp and sunflower than in maize (**Supplementary Figure 4**, panel 1), reflecting fundamental species-specific differences in cell wall composition, root anatomy, and transpiration-driven uptake. Dose-dependent reductions in root growth and changes in photosynthetic parameters under PFAS exposure in hydroponically grown maize further support that maize root physiology is sensitive to perfluorinated compounds, even when aboveground growth is maintained (Ebinezer et al., 2022).

Hemp was especially effective at accumulating PFOA aboveground, reaching a leaf concentration of 5.39 μg g^-1^ d.w. and a BCF_l_ of 10.78. This BCF indicates relatively strong PFOA accumulation in hemp leaves under the tested pot conditions, supporting hemp as a species of interest for further PFAS phytoextraction research (Nassazzi et al., 2023). However, BCF values were lower when calculated for the whole aboveground biomass (leaf + stem; BCF_ab_). This was because BCF_ab_ is a biomass−weighted mean of leaf and stem concentrations. The inclusion of stems, which contained much lower PFOA concentrations than leaves, reduces the overall aboveground BCF relative leaves. Hemp’s root architecture, characterized by a deep taproot and abundant lateral roots (Amaducci et al., 2008), together with high transpiration rate, may have contributed to its high shoot accumulation by enhancing contaminant interception and xylem transport (Tang et al., 2018).

Although both PFOA and PFOS are long-chain C8 PFAS, their accumulation patterns differed among species. Maize retained proportionally more PFAS in roots, whereas hemp and sunflower showed stronger translocation, especially for PFOA (Birchfield et al., 2025; Zhang et al., 2019). Maize roots have a phenolic-rich, pectin-poor Type II cell walls, rich in glucuronoarabinoxylan cross-linked by ferulic and *p*-coumaric acids, which can strongly adsorb hydrophobic long-chain PFAS in the root apoplast, acting as a barrier to upward movement. In contrast, hemp and sunflower have pectin-rich Type I cell walls, which are more porous and with high hydraulic conductivity, potentially enhancing PFAS xylem and shoot translocation (Carpita and Gibeaut, 1993). As a result, maize’s specific wall composition most likely contributes to its restricted PFAS transfer to aerial tissues.

Our soil-based experiment showed higher PFOA than PFOS accumulation in hemp leaves (5.39 vs 2.59 µg g^-1^ d.w.), which contrasts with findings from *in-vitro* grown hemp where PFOS accumulation exceeded PFOA (43.7 vs. 7.8 µg g^-1^ d.w.; Luche et al., 2026a). This discrepancy likely reflects the soil matrix effects: in our silty-loam soil with 1.77% organic matter, PFOS’s sulfonate group associates more strongly with soil humic substances than PFOA’s carboxylate group, thereby reducing PFOS mobility and plant uptake, and aboveground translocation (Milinovic et al., 2015; Costello and Lee, 2020). In a soilless medium, PFOS remains more available for uptake, which would explain stronger accumulation *in vitro* found by Luche et al. (2026a). These differences highlight the importance of considering the growth substrate, exposure system, together with plant age when comparing PFAS uptake across studies.

A clear trade-off exists between high tissue concentration and total PFAS removal per plant. Hemp showed higher concentrations of PFAS in leaf tissues, while maize and sunflower showed comparatively better total accumulation per plant due to their superior individual plant biomass output (He et al., 2023). This is consistent with Nassazzi et al. (2023), who demonstrated that biomass production can outweigh tissue PFAS concentration in determining a species-level removal capacity. PFOA was generally accumulated to a greater extent than PFOS in leaves across species (Huff et al., 2020; He et al., 2023; Wen et al., 2016). Greater root retention of PFOS relative to PFOA is consistent with its greater lipophilicity, stronger binding to humic substances, and the role of the Casparian strip in restricting hydrophobic long-chain compound movement to the shoot (Wang et al., 2020; Milinovic et al., 2015).

PFAS accumulation values reported here refer to individual plants, and plant density differed among species, with five plants per pot in hemp and three in maize and sunflower. Therefore, the greater PFAS burden per plant observed in maize and sunflower partly reflects both species-specific growth and the lower within-species competition imposed by the pot design. These per-plant removals do not directly represent field-level removal capacity at agronomically relevant planting densities. In agronomic practice, hemp is generally cultivated at relatively high stand densities, reaching approximately 100 to 250 plants m^-2^, with 180 to 270 plants m^-2^ commonly recommended for fiber hemp production (Amaducci et al., 2002), compared with 5 to 10 plants per m^-2^ for sunflower and maize. Therefore, per-area PFAS extraction of hemp may be underestimated by per-plant comparisons alone. This point reinforces the need for field-scale evaluation at realistic sowing densities and with PFAS removal expressed per unit area. Moreover, the use of non-perforated pots, adopted to prevent PFAS leaching for safety reasons, precluded free drainage and may have altered rhizosphere conditions relative to field-drained soils, although irrigation was managed to avoid prolonged waterlogging. The relatively small pot volume (1.7 L) should also be considered when interpreting absolute PFAS uptake and translocation values in the context of open−field conditions; however, no visible waterlogging symptoms were observed during and at the end of the trial (**Supplementary Figure 2**).

From an application standpoint, bioaccumulation of PFAS in roots is less relevant, since only aboveground biomass is practically harvestable for disposal and roots are usually left in the ground. In plant species with low translocation factors, especially maize and sunflower, a substantial fraction of the absorbed PFAS therefore remains in the soil-root zone. Accordingly, root retention should be interpreted more as phyto-stabilization than as effective phytoextraction.

Several experimental studies report that PFAS concentrations are often higher in roots than in shoots, reinforcing the dominance of root sequestration processes (Kenyon et al., 2024; Xu et al., 2022; Qian et al., 2023; Wang et al., 2020). Root retention of PFAS may be advantageous in remediation scenarios where contaminant containment and reduced off-site transport are the primary objectives, as organic matter amendments (including humic acids and compost) can reduce PFAS bioavailability and mobility in the soil–plant system (Liu et al., 2023; Li et al., 2020). However, this shift is less favorable when the objective is phytoextraction, which relies on efficient translocation of contaminants into harvestable aboveground biomass. Indeed, multiple studies emphasize that PFAS phytoextraction efficiency is generally limited by restricted root-to-shoot transfer, particularly for hydrophobic and long-chain compounds (Nassazzi et al., 2023). The long-term stability of root-retained PFAS warrants further investigation. Root-associated PFAS may be remobilized during root decay or transformed within the rhizosphere (Costello and Lee, 2020), and post-harvest mass balance monitoring will be needed to determine whether root-retained PFAS remain stabilized over time.

Removal efficiency of aboveground biomass was measurable but modest across all species. The highest removal was approximately 2% in hemp, which falls within the range reported for greenhouse pot studies of PFAS phytoremediation, where removal efficiency ranged from 0.04% to 41.4% across species and compounds, with lower values reported for long-chain PFAS such as PFOA and PFOS (He et al., 2023). Similar low efficiencies have also been reported for hemp in pot-scale experiments (Nassazzi et al., 2023). These low efficiencies likely reflect the early vegetative growth stage (32 DAS), the limited plant density adopted in pots, and the modest soil volume available for root exploration, rather than the full phytoremediation potential of the tested species under field conditions.

### 4.3 Effects of humic acids soil application

Soil application of humic acids produced more evident effects on plant growth than on PFAS accumulation. However, interpretation of this treatment requires caution because the product used in this study (Humic Super Bio) was a commercial leonardite-derived humic formulation for which the manufacturer reports KOH as the extracting agent. Because the soil was already alkaline at the start of the experiment (pH 8.15; **Supplementary Table 1**), large shifts in bulk soil pH were expected to be limited due to the buffering capacity typically associated with alkaline soils (Han et al., 2023). However, PFAS partitioning is strongly influenced by soil pH and interactions with soil organic matter and charged soil surfaces (Campos-Pereira et al., 2023; Uwayezu et al., 2022). Since neither product pH nor post-application soil pH was measured, and no KOH-only control was included, an independent contribution of formulation chemistry cannot be excluded.

In uncontaminated soil, HAs slightly stimulated growth and increased biomass by 2% in hemp, 17% in maize and 20% in sunflower relative to the controls. The significant 49% increase in sunflower root biomass under HAs alone suggests that, in this species, the most pronounced organ-level response to the commercial humic formulation used here was expressed belowground under non-contaminated conditions. This pattern is consistent with the well-documented biostimulant action of humic substances on root development and nutrient uptake (Nardi et al., 2002; Conselvan et al., 2017; Canellas and Olivares, 2014; Muscolo et al., 2013). Statistically significant HAs effects were limited to a small number of growth-related traits, including total dry weight in maize and sunflower, sunflower root biomass, and SPAD in maize at 28 DAS. However, because root architectural and physiological traits were not measured directly, this interpretation should be regarded as a functional explanation of the observed biomass pattern rather than as evidence of a specific mechanism.

HA-related effects on PFAS tissue concentration and total plant PFAS burden were generally non-significant, with slight improvements for PFOS only, although in hemp TF was lower under PFAS+HAs than under PFAS for both PFOA and PFOS. This limited response may also reflect application sequence, because PFAS were applied at 8 DAS and HAs at 14 DAS, so the PFAS+HAs treatment represented HAs addition to an already PFAS-exposed soil-plant system rather than simultaneous co-application. This temporal offset may have reduced early HAs–PFAS interactions in the soil phase and thereby limited their influence on PFAS bioavailability and subsequent root uptake (Milinovic et al., 2015; Qi et al., 2022).

Under PFAS contamination, the growth-promoting effect of HAs was less evident and, in some cases, negative. For example, sunflower under PFAS+HAs showed a 10% reduction in total plant biomass relative to the control. In hemp, HAs shifted PFAS partitioning toward roots, with lower concentrations in leaves than under PFAS alone. This non-significant pattern may be consistent with the hypothesis that humic substances promoted the formation of PFAS-DOM (dissolved organic matter) complexes or greater root-zone PFAS retention, thereby reducing translocation to shoots (Qi et al., 2022; Chianese et al., 2020). However, this interpretation is speculative in the absence of DOM characterization and soil PFAS fraction data. In contrast, this effect was less evident in maize and sunflower, and did not reach statistical significance.

The addition of HAs to PFAS-contaminated soil also impacted the uptake of PFOA and PFOS differently. With HAs in the soil, the total PFOA concentration in plants tended to decrease, while total PFOS concentration slightly increased, regardless of the species considered (**Supplementary Figure 4**, panel 1). This result is counterintuitive given PFOS’s stronger binding affinity for humic substances, which would reduce its bioavailability more than PFOA’s (Milinovic et al., 2015). One hypothesis is that root-DOM-PFOS complexes formed more readily than root-DOM-PFOA complexes, promoting PFOS retention in roots. Alternative mechanisms cannot be excluded: the KOH-extracted commercial formulation and the sequential application of PFAS and HAs may both have altered root-zone PFAS sorption-desorption behavior and PFAS-soil-root interactions (Campos-Pereira et al., 2023; Qi et al., 2022). Because neither post-application soil pH nor soil-phase PFAS fractions were measured, these competing mechanisms cannot be distinguished at this time.

Overall, under the tested conditions, a single soil application of this commercial humic formulation at 1 g kg^-1^ acted primarily as a growth biostimulant, with only limited influence on PFAS accumulation and translocation. Therefore, its role in PFAS phytoremediation should be considered preliminary until dose-response relationships and soil chemistry interactions are characterized more broadly.

## 5 Conclusion

Over one month of growth in soil spiked with PFOA and PFOS at 500 µg kg^−1^ each, hemp, maize, and sunflower maintained whole-plant biomass and leaf chlorophyll without visible phytotoxicity. This indicates that the tested PFAS exposure was sub-lethal at the whole-plant level during early vegetative stage tested here. However, this should not be interpreted as the absence of physiological stress. In hemp, PFAS exposure increased PSII operating efficiency while significantly reducing stomatal conductance, transpiration, and net CO_2_ assimilation and ETR, revealing a decoupling between photochemical function and carbon fixation. This physiological response is a novel finding, underlining that biomass-based endpoints alone may underestimate PFAS stress at least in hemp. Therefore, this signal warrants further investigation to clarify the links among PFAS exposure, stomatal regulation, and photosynthetic performance, including in other species, such as the C3 sunflower, and particularly the C4 maize, which has a different photosynthetic pathway. Repeated gas-exchange and chlorophyll-fluorescence monitoring across plant development will also be essential to define the onset, persistence, and possible recovery of PFAS effects.

Among the three species tested here, hemp showed the strongest root-to-shoot translocation and the highest foliar PFAS concentrations, particularly for PFOA, indicating a greater capacity to concentrate PFAS in harvestable aboveground tissues under the tested conditions and supporting its potential use in phytoremediation programs. However, species ranking may differ when based on tissue concentration vs. whole−plant contaminant mass removal, as maize and sunflower achieved considerable total plant PFAS burden because of their greater biomass. Complete phytoremediation potential assessments require moving from mesocosm to open field systems under real environmental conditions. Continued phytoextraction research on hemp is advisable, although its practical relevance will depend on agronomic optimization and long-term field validation across different soils, crop cycles, and plant densities. These advances would improve both PFAS-specific phytoremediation strategies and food-safety risk assessment. The long-term fate of PFAS in the root zone remains an open question, and studies covering full crop cycles, combined with soil mass balance monitoring, would also help determine the persistence and mobility of PFAS in the soil-plant and aquatic systems over time.

Humic acids demonstrated their primary role as plant growth biostimulants, with clearer effects on biomass production than on PFAS accumulation or partitioning. While HAs tended to shift PFAS partitioning toward roots, likely due to their binding interactions with these pollutants, their role in PFAS phytoremediation should be considered preliminary, and different methods of application, such as foliar spraying, should be tested to limit their possible neutralization at soil-root level.

The present results at 500 µg kg^−1^ per PFAS compound establish a useful reference point on the dose-response curve, because of the sub-lethal effects in all three plant species. Multi-dose experiments will be needed to establish dose-response relationships and species-specific sensitivity thresholds, including half−maximal inhibitory concentration (IC50), and half−maximal effective concentration (EC50) values. In parallel, integrating antioxidant enzyme activity, oxidative damage markers, PFAS localization, and other biochemical endpoints would clarify whether humic acids mainly modulate plant stress responses or alter PFAS uptake and internal distribution.

## Data Availability

The raw data supporting the conclusions of this article will be made available by the authors, without undue reservation.
