# Supplementary material for: PFAS-induced morpho-physiological, photosynthetic and tissue accumulation responses of pot-grown hemp, sunflower and maize under soil amendment with humic acids
Source: Front Plant Sci. 2026 Jul 7;17:1791472. doi: 10.3389/fpls.2026.1791472 (PMC13384836; doi:10.3389/fpls.2026.1791472)
Supplement: Supplementary file 1 [file Supplementaryfile1.docx]

Supplementary Material

**Table S1**: Key physical and chemical properties of the soil used as pot substrate for the trial.

| **Property** | **Value** |
| --- | --- |
| Silt (%) | 65 |
| Sand (%) | 15 |
| Clay (%) | 20 |
| pH | 8.15 |
| Organic Matter (%) | 1.77 |
| N (%) | 0.11 |
| C/N | 9.72 |
| CEC (cmol (+) kg^−1^) | 15.4 |
| Total P (mg P_2_O_5_ kg^−1^) | 810 |
| Available P (mg P_2_O_5_ kg^−1^) | 8.46 |
| Exchangeable K (mg K_2_O kg^−1^) | 59.9 |
| Exchangeable Mg (mg kg^−1^) | 247 |
| Exchangeable Ca (mg kg^−1^) | 2,619 |
| Exchangeable Na (mg kg^−1^) | 26.1 |
| Total S (mg kg^−1^) | 408 |

**Figure S1**: Dynamics of foliar chlorophyll content, expressed as Soil Plant Analysis Development (SPAD) values, in hemp (mean±S.E.; n=6), maize and sunflower plants (mean±S.E.; n=3) under CTRL (control), HAs (humic acids), PFAS (soil contamination), and PFAS+HAs (soil contamination + humic acids) treatments. Within each date (DAS, Days After Sowing), asterisks indicate significant differences among treatments (* *p*≤0.05; ** *p*≤0.01; *** *p*≤0.001; n.s.=not significant). Letters: statistical comparison among treatments within the same date (Tukey’s test, *p*≤0.05).


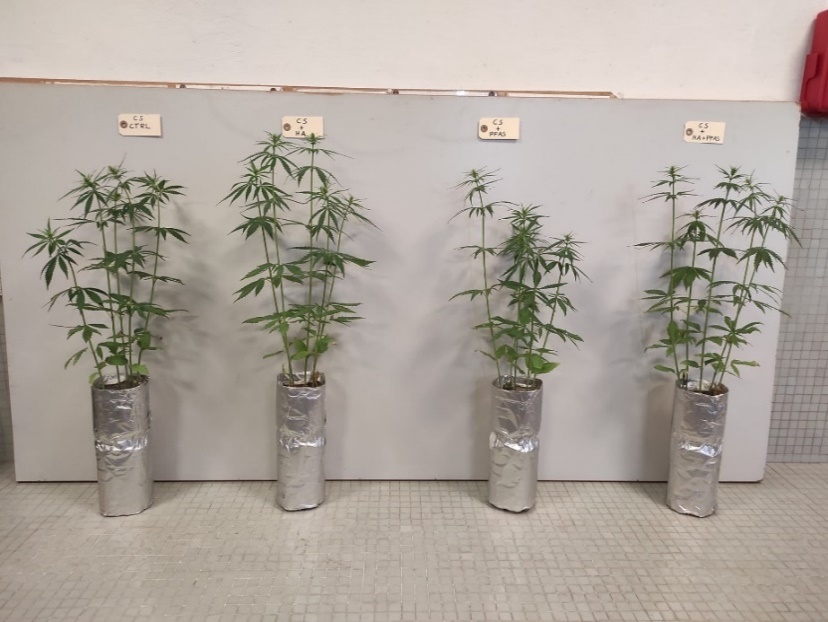


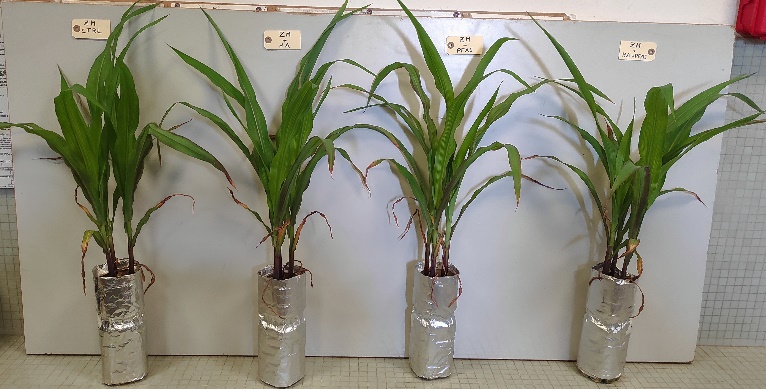


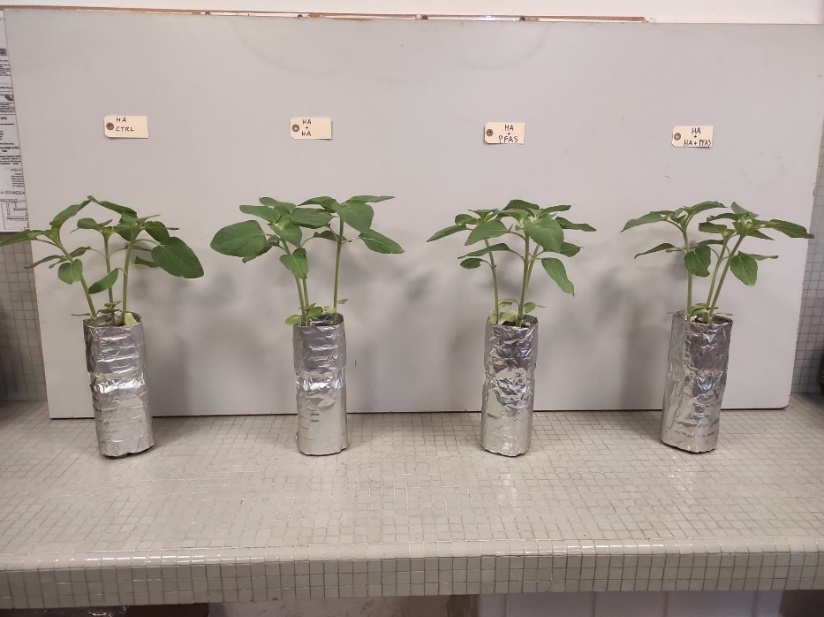


**Figure S2**: Visual representation of treatments at the end of the experiment (32 DAS). From left to right: CTRL (control), HAs (humic acids), PFAS (soil contamination), and PFAS+HAs (soil contamination + humic acids) in hemp (top), maize (middle) and sunflower (bottom).

**Figure S3**: Dynamics of shoot height in hemp (mean±S.E.; n=6), maize and sunflower (mean±S.E.; n=3) under CTRL (control), HAs (humic acids), PFAS (soil contamination), and PFAS+HAs (soil contamination + humic acids) treatments. Within each date (DAS, Days After Sowing), asterisks indicate significant differences among treatments (* *p*≤0.05; ** *p*≤0.01; *** *p*≤0.001; n.s. = not significant). Letters: statistical comparison among treatments within the same date (Tukey’s test, *p*≤0.05).

**Figure S4:** Panel 1 shows PFOA, PFOS and PFOA+PFOS concentrations in total plant biomass of hemp (CS) (mean±S.E.; n=6), maize (ZM) and sunflower (HA) (mean±S.E.; n=3), and panel 2 shows PFOA+PFOS concentrations in leaves, stems and roots as an average of the three species (main effect) (mean±S.E.; n=12) under PFAS (soil contamination) and PFAS+HAs (soil contamination + humic acids) treatments. Percentages: variations vs. reference (Ref.). Letters: statistical comparison within the factor under study namely treatment in Panel 1 and tissue Panel 2, (Tukey’s test, p≤0.05).

**Figure S5**: Aboveground biomass bioconcentration factor (BCF) of PFOA, PFOS and their sum in hemp (mean±S.E.; n=6), maize and sunflower plants (mean±S.E.; n=3), under PFAS (soil contamination) and PFAS+HAs (soil contamination + humic acids) treatments. BCF values are reported above bars. Letters: statistical comparison among PFOA, PFOS and PFOA+PFOS (Tukey’s test, *p* ≤ 0.05). The horizontal dashed red line indicates reference line at BCF=1.

**Figure S6**: Panel 1 shows the aboveground plant biomass removal efficiency (%) and panel 2 shows the total plant biomass removal efficiency (%) of PFOA, PFOS and PFOA+PFOS in hemp (CS) (mean±S.E.; n=6), maize (ZM) and sunflower (HA) plants (mean±S.E.; n=3) grown in a pot, under PFAS (soil contamination) and PFAS+HAs (soil contamination + humic acids) treatments. Percentages: variation vs. hemp (Ref.). Letters: statistical comparison among plant species (Tukey’s test, *p* ≤ 0.05).
